# Recognizing patient partner contributions to health research: a systematic review of reported practices

**DOI:** 10.1186/s40900-023-00488-5

**Published:** 2023-09-09

**Authors:** Grace Fox, Manoj M. Lalu, Tara Sabloff, Stuart G. Nicholls, Maureen Smith, Dawn Stacey, Faris Almoli, Dean A. Fergusson

**Affiliations:** 1 School of Epidemiology and Public Health, University of Ottawa, Ottawa, ON Canada; 2 Clinical Epidemiology Program, Ottawa Hospital Research Institute, Ottawa, ON Canada; 3 Department of Cellular and Molecular Medicine, University of Ottawa, Ottawa, ON Canada; 4 Department of Anesthesiology and Pain Medicine, University of Ottawa, The Ottawa Hospital, Ottawa, ON Canada; 5 Office for Patient Engagement in Research Activities (OPERA), Ottawa Methods Centre, Ottawa Hospital Research Institute, Ottawa, ON Canada; 6 Patient partner, Ottawa, ON Canada; 7 University of Ottawa, School of Nursing, Ottawa, ON Canada; 8 Department of Medicine, University of Ottawa, Ottawa, ON Canada

**Keywords:** Patient engagement, Patient partner, Recognition, Financial compensation

## Abstract

**Background:**

Patient engagement in research refers to collaboration between researchers and patients (i.e., individuals with lived experience including informal caregivers) in developing or conducting research. Offering non-financial (e.g., co-authorship, gift) or financial (e.g., honoraria, salary) compensation to patient partners can demonstrate appreciation for patient partner time and effort. However, little is known about how patient partners are currently compensated for their engagement in research. We sought to assess the prevalence of reporting patient partner compensation, specific compensation practices (non-financial and financial) reported, and identify benefits, challenges, barriers and enablers to offering financial compensation.

**Methods:**

We conducted a systematic review of studies citing the Guidance for Reporting the Involvement of Patients and the Public (GRIPP I and II) reporting checklists (October 2021) within Web of Science and Scopus. Studies that engaged patients as research partners were eligible. Two independent reviewers screened full texts and extracted data from included studies using a standardized data abstraction form. Data pertaining to compensation methods (financial and non-financial) and reported barriers and enablers to financially compensating patient partners were extracted. No formal quality assessment was conducted since the aim of the review is to describe the scope of patient partner compensation. Quantitative data were presented descriptively, and qualitative data were thematically analysed.

**Results:**

The search identified 843 studies of which 316 studies were eligible. Of the 316 studies, 91% (n = 288) reported offering a type of compensation to patient partners. The most common method of non-financial compensation reported was informal acknowledgement on research outputs (65%, n = 206) and co-authorship (49%, n = 156). Seventy-nine studies (25%) reported offering financial compensation (i.e., honoraria, salary), 32 (10%) reported offering no financial compensation, and 205 (65%) studies did not report on financial compensation. Two key barriers were lack of funding to support compensation and absence of institutional policy or guidance. Two frequently reported enablers were considering financial compensation when developing the project budget and adequate project funding.

**Conclusions:**

In a cohort of published studies reporting patient engagement in research, most offered non-financial methods of compensation to patient partners. Researchers may need guidance and support to overcome barriers to offering financial compensation.

## Abstract (plain language summary)

The term *patient engagement in research* is used to describe research that is conducted “with” patients, rather than “on” patients. It is important that researchers recognize patient partners for their time and expertise. In order to gain a better understanding of approaches to recognition for patient partners we reviewed published studies to: (1) assess how often financial compensation is reported, (2) identify how patient partners are reported as being compensated, and (3) understand what benefits, challenges, barriers and enablers might exist to offering financial compensation. We conducted a systematic review of articles citing the Guidance for Reporting the Involvement of Patients and the Public (GRIPP) guidelines. We included all study designs if patients were engaged as partners. Studies in which patients were participants only were excluded. Data collected included information about details of patient partner compensation (financial and non-financial practices) as well as challenges relating to financial compensation. Numerical data were analysed descriptively. Textual data were coded by two reviewers and collated into overarching themes. Our search identified 316 papers. Of these, 91% reported offering compensation to patient partners. Most common methods were acknowledgement (65%) and co-authorship (49%). Only 79 studies (25%) reported offering financial compensation to patient partners. Limited funding and lack of institutional guidance were identified as two key barriers that may be preventing researchers from offering financial compensation. Our review found that non-financial methods of compensation are reported more often than financial compensation. Researchers may require more support when offering financial compensation to patient partners.

## Introduction

Patient engagement in research—also commonly referred to as a patient and public involvement [1] or patient and public engagement [2]—refers to the active inclusion of individuals with lived experience of a health issue, including informal caregivers, family and friends [3], in the research process [4]. It is research carried out ‘with’ patients and not ‘on’, ‘about’ or ‘for’ them [5]. Patient engagement in research yields numerous benefits including improved study quality, better clinical trial recruitment, and alignment of research priorities with needs of the ultimate end user [6]. As a result, there is growing advocacy for patient engagement in health research [6–8].

Recognizing patient partner contributions by offering non-financial or financial compensation has been proposed as a strategy to encourage continued patient engagement and demonstrate that patient partner efforts are valued [9]. Feeling valued is crucial to fostering an inclusive team atmosphere, which plays a pivotal role in supporting sustained and active patient engagement in research [10, 11]. Patient partner recognition methods can be non-financial (e.g., co-authorship) or financial (e.g., honoraria). Financial compensation, in particular, can serve as a facilitator by addressing important barriers to engagement [12]. Without financial compensation, only patient partners with time and resources will be able to become patient partners. To support researchers in developing compensation strategies, several patient-oriented organizations have developed guidance documents encompassing both non-financial and financial methods [13–16]. In addition, some funding agencies have established guidelines to assist applicants in budgeting for engagement [15–21]. Despite available guidance and policies, little is known regarding how researchers recognize patient partners either non-financially or financially. Similarly, researcher attitudes on financial compensation including benefits, challenges, barriers and enablers remain unclear.

Given this significant knowledge gap, the aim of our systematic review was to answer the following research questions: *How are researchers compensating patient partners for their contributions to research? What is the prevalence of reporting patient partner financial compensation?* and *What are researcher attitudes towards patient partner financial compensation, including perceived benefits, challenges, barriers and enablers to offering financial compensation?* To address these research questions we assessed the prevalence of reporting patient partner compensation among published research that engaged patients as well as identified non-financial and financial methods of compensation, the monetary values of financial compensation, and any guidance documents reported as informing the approaches. The review findings provide a contemporary overview of compensation strategies to help researchers, and inform implementation strategies to better support patient partner compensation.

## Methods

Our systematic review was conducted in accordance with methodology detailed in the Cochrane Handbook for Systematic Reviews of Interventions [22] and this report is prepared in accordance with the Preferred Reporting Items for Systematic Reviews and Meta-analyses (PRISMA) reporting guidelines (Appendix 1) [23]. The protocol was published as part of a larger research program [24] and can be found on the International Prospective Register of Systematic Reviews (PROSPERO ID: CRD42022303226). A patient partner (MS) was engaged in this study and engagement activities are reported using the Guidance for Reporting the Involvement of Patients and the Public (GRIPP2) [25] checklist (Appendix 2).

### Search strategy and information sources

We identified studies that cited the Guidance for Reporting the Involvement of Patients and the Public (GRIPP 1 and 2) [25, 26] checklists to report engagement activities and outcomes. Using this forward citation search, we hypothesised that we were more likely to capture a cohort of studies that had engaged patients as partners than we would have with a broader search filter; previous studies have demonstrated a very low rate of reporting patient engagement in research overall [27], thus the specificity of a broad literature search would be exceedingly low rendering the review infeasible. Consequently, we chose studies that reference GRIPP or GRIPP2 as a specific, efficient, and targeted search strategy to identify a cohort of published studies that had engaged patients. The forward citation search was conducted using the Scopus and Web of Science databases on October 14, 2021. An information specialist (Lindsey Sikora, Health Sciences Research Librarian, University of Ottawa) was consulted when developing the forward citation search strategies. All included studies were necessarily published after the GRIPP I publication date (2011).

### Eligibility criteria

We included studies that were written in English, reference the GRIPP checklists (I or II) [25, 26] and engaged or described engaging patients in health research, in which members of the public or patients (i.e., an individual with lived experience of a health condition as well as informal caregivers, including family, friends, or members of patient organizations) are engaged as partners (provided input, guidance, consultation on at least one element of the research process) [4].

Studies that engaged patients as partners as well as participants (i.e., subjects of research) were included in the review, while studies where patients were involved solely as research participants were excluded. Examples of research engagement include priority-setting, governance, developing the research question, identifying study outcomes, study design, informing statistical analysis, interpreting study findings and disseminating results [28]. We included all study designs (e.g., qualitative, quantitative, evidence synthesis, mixed-methods research). Studies that described the patient engagement in research component as a part of a larger research project were included. Studies that interviewed patients about their experiences as patient partners were excluded as patients were participants in the research [29]. Conference abstracts and commentaries were also excluded.

### Selection process

All records identified by the literature search were uploaded to DistillerSR (a cloud-based software program that facilitates systematic reviews) (Evidence Partners Incorporated, Ottawa, Canada) [30]. After duplicate removal, reviewers (GF, TS, FA) independently screened full-text articles according to the pre-specified eligibility criteria. The screening process was piloted for the first 20 articles to ensure reliability between reviewers. A third reviewer (DAF or MML) was consulted if reviewers could not reach consensus. Reasons for exclusion were recorded using the PRISMA flow diagram.

### Data collection process

Two reviewers (GF, TS) independently extracted data from studies included in the systematic review using a data extraction form in DistillerSR. Data extraction was piloted for the first five studies to ensure consistency. After piloting, the reviewers extracted data from sets of 20 studies and resolved conflicts between each set. A third reviewer (DAF or MML) was consulted if reviewers could not reach consensus. We did not contact authors for missing or additional information since the focus of this systematic review was on the reporting of patient partner compensation practices.

### Data items

Extracted items included study characteristics (e.g., author information, source of funding), patient engagement characteristics (e.g., level of engagement, type of stakeholder engaged), details of patient partner compensation (e.g., non-financial and financial practices), and any reported benefits, challenges, barriers and enablers to financial compensation. The country of the corresponding author’s institutional location was also extracted. For the purposes of this review, we defined non-financial compensation as offering tokens of appreciation or services in exchange for patient partnership on a research project, and financial compensation as offering something of direct monetary value in exchange for their involvement [19, 31, 32]. Gifts or gift cards were considered financial compensation only when the value was informed by a formal conversion (i.e., 2 h of work at $25 per hour = $50 gift or gift card value) or where they were reported as being given as a substitute for monetary payment based on the work undertaken. Financial compensation practices were identified based on reports with no initial list of categories. Rather, categories were developed inductively based on the developing list of items. For example, financial compensation based on a fixed monetary value (irrespective of workload) was defined as honoraria. While reported variously as being paid cash, cheques or stipend, these approaches were grouped under the category of honoraria. Studies that explicitly reported offering a salary to patient partners were grouped under the category of salary. Patient engagement activities were categorized as *Consult, Collaborate*, and *Empower* levels of engagement in accordance with definitions developed by the National Institute for Health and Care Research (NIHR) [33]. It is important to note that studies could achieve more than one level of engagement if different activities took place at different stages of the same research study. Engagement activities were categorized as occurring at different stages of the research process (e.g., study design, data collection, data analysis), governance (i.e., member of a committee overseeing the research project), general priority setting (i.e., identifying research priorities to inform future research) or general outcome derivation (i.e., identifying outcomes to be captured in future studies). A full list of data extraction items can be found in Appendix 3.

### Study risk of bias assessment

No formal quality assessment was conducted for included studies since the aim of the review is to describe the use and type of patient partner compensation.

### Synthesis methods

Patient engagement and compensation details (e.g., stakeholders engaged, length of engagement, type of financial compensation offered) were created inductively and analyzed descriptively. Prevalence of reporting patient partner financial compensation was calculated. Qualitative data (reported benefits, challenges, barriers and enablers to patient partner compensation) were analyzed thematically in accordance with the 6-step approach developed by Braun and Clark [34]. Verbatim statements were extracted by two independent reviewers (GF, TS) and stored in an Excel file. Two independent reviewers (GF, SN) read through extracted verbatim statements and generated initial codes within each of the five domains (benefits, challenges, barriers, enablers, justification). Reviewers met regularly to resolve discrepancies between initial codes. Initial codes were collated into overarching themes and reoccurring themes were combined. The frequency of reported themes was recorded.

### Subgroup analysis

Prespecified subgroup analyses of reporting patient partner financial compensation were performed according to funding (funded vs. non-funded) and level of engagement (*Consult, Collaborate, Empower*) [35]. Given that levels of engagement are ordered based on the level of impact that patient partners have on decision making, we compared studies based on the highest level of engagement reported. For example, studies that engaged patient partner’s at all three levels of engagement were in the same subgroup as studies that only engaged patient partners at the *Empower* level of engagement since both achieved *Empower* as the highest level of engagement. A post hoc subgroup analysis by country (comparing studies conducted in the United States, Canada and the United Kingdom with the remaining studies) was conducted following consultation with a group of patient partners (i.e., Ontario SPOR Support Unit Patient Partner Working Group). This was based on the hypothesis that studies conducted in the United States, Canada and the United Kingdom may be more likely to practice patient partner compensation given support from well-established national infrastructures (i.e., Patient Centered Outcomes Research Institute (PCORI), Canadian Institutes of Health Research (CIHR), and National Institutes for Health and Care Research (NIHR) respectively). Subgroup proportions were compared using Chi-square tests.

### Patient and public involvement

One patient partner (MS) informed project development (e.g. review proposals and protocols, identifying sources, research question generation) and provided feedback on project conduct (e.g., data extraction, interpretation). MS has a wealth of experience with various facets of patient engagement in research including experience with various methods of compensation. Monthly meetings occurred with MS to discuss research findings as the systematic review progressed. We co-developed a terms of reference document a priori to establish details of engagement (e.g., expectations, project goals, compensation). Our patient engagement in research plan was informed by INVOLVE’s Seven Core Principles of Engagement [36] and the CIHR Strategies for Patient Oriented Research (SPOR) Patient Engagement framework [3]. Co-authorship and financial compensation were agreed upon with the patient partner and offered as a method of acknowledgement according to the SPOR Evidence Alliance Patient Partner Appreciation Policy [13]. The aim of collaboration was to ensure that the patient perspective was considered throughout the project.

## Results

### Search results

**Fig. 1:**
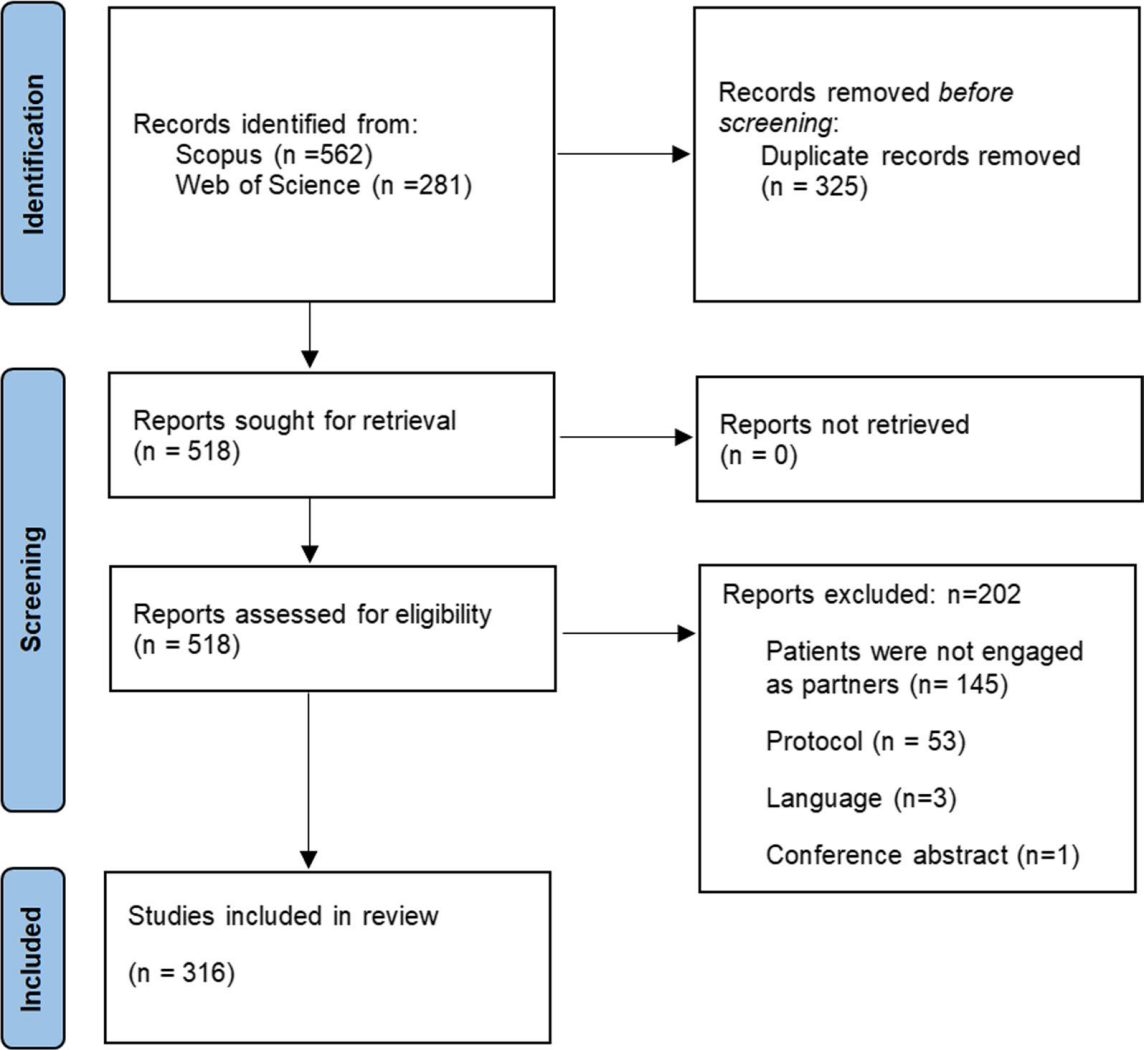
PRISMA flow diagram

Our search retrieved 843 studies. After removing duplicates, 518 studies were screened and assessed for eligibility and a total of 316 studies were included in the systematic review. Screening results are presented using a PRISMA flow diagram (Fig. 1). A full list of included studies can be found in Appendix 4.

### Study characteristics

Most corresponding authors were based in the United Kingdom (60%, n = 190) followed by Canada (16%, n = 51) and Denmark (5%, n = 15) (Appendix 5). Only one corresponding author was based in a Low- or Middle Income Country (LMIC) (South Africa). The earliest study was published in 2011 and the largest proportion of studies was published in 2020 (27%, n = 84), followed by 2021 (24%, n = 77) (Appendix 6). Most studies (84%, n = 265) were funded and 33 (12%) funded studies reported receipt of funding specifically to support patient engagement (Appendix 7A). Most funded studies received funding from government agencies (75%, n = 200) (Appendix 7B).

### Patient engagement in research characteristics

Studies reported engaging a variety of stakeholders including patients (78%, n = 246), caregivers (35%, n = 111), and members of patient organizations (16%, n = 50) (Table 1). A median of five patient partners were reported for each study with a range of 1–705 patient partners. The study reporting 705 patient partners detailed three engagement efforts, including a conference event attended by patient partners [37].

**Table 1:** Patient partner characteristics of studies (n = 316)

Patient partner characteristics	Number of studies (%)
*Type of stakeholder engaged*	
Patient	246 (78)
Caregivers	111 (35)
Member of patient organizations	50 (16)
Community member	46 (15)
Family member	25 (8)
Friends	1 (0.3)
Not specified	25 (8)
*Number of patients engaged*	
1	31 (10)
2–5	84 (26)
6–10	57 (18)
11–36	72 (23)
40–70	12(4)
100+	13 (4)
Not specified	47 (15)
*Were patient partners engaged more than once?*	
No	249 (79)
Yes	55 (17)
Unclear	12 (4)

Studies described engagement occurring at various stages across the research process including governance (24%, n = 76), funding acquisition (17%, n = 54), priority setting (17%, n = 52), study design (79%, n = 250), data collection (14%, n = 45), data analysis (43%, n = 137), dissemination of results (50%, n = 157), developing patient engagement plan (8%, n = 24), and ethics application development (0.3%, n = 1) (Table 2).We identified studies that engaged patient partners in general priority setting exercises (13%, n = 42) and general outcome derivation (5%, n = 16). Level of engagement in research reported were *Consult* (66%, n = 204), *Collaborate* (53%, n = 163), and *Empower* (14%, n = 44). We identified six studies (2%) that engaged patient partners at all three levels.

**Table 2:** Patient engagement characteristics of studies (n = 316)

Patient engagement characteristic	Number of studies (%)
*Stage of research where patient partners contributed*	
Study design	250 (79)
Dissemination of results	157 (50)
Data analysis	137 (43)
Funding	54 (17)
Priority setting	52 (17)
Data collection	45 (14)
Developing patient engagement strategy	24 (8)
Ethics	11 (3)
Governance	76 (24)
General priority setting	42 (13)
General outcome derivation	16 (5)
Not reported	5 (2)
*Level of engagement*	
Consult	111 (35)
Collaborate	73 (23)
Empower	19 (6)
Consult and Collaborate	76 (24)
Consult and Empower	11 (3)
Collaborate and Empower	8 (3)
Consult and Collaborate and Empower	6 (2)
Activities are unclear	12 (4)

### Reporting non-financial compensation practices

Of the 316 studies, 91% (n = 288) reported offering some type of compensation (i.e., non-financial or financial compensation) to patient partners. The most common reported method of non-financial compensation was informal acknowledgement on research outputs (e.g., acknowledgement section in publications, dissemination documents, presentations) (65%, n = 206) and co-authorship (49%, n = 156) (Table 3). Additional methods of non-financial compensation included facilitating patient partner attendance at conferences (7%, n = 2) and offering training opportunities (12%, n = 4). Twenty-eight (9%) studies reported not offering any form of non-financial compensation to patient partners.

**Table 3:** Non-financial methods of compensating patient partners (n = 316)

Non-financial compensation	Number of studies (%)
Acknowledgements	206 (65)
Co-authorship on a manuscript	156 (49)
Reimbursement of expenses incurred from engagement	67 (21)
Provided meals	37 (12)
Listed as a co-investigator or co-applicant	33 (10)
Conference presenter	23 (7)
Conference attendance	12 (4)
Provided transportation	9 (3)
Certification or training opportunities	7 (2)
Token of appreciation (i.e., gift)	4 (1)
Gift card	4 (1)
Scholarship	2 (0.6)
Providing babysitting services	1 (0.3)
None	28 (9)

Multiple methods of non-financial compensation can be reported in a single study

### Reporting financial compensation practices

Of the 316 studies, 25% (n = 79) reported offering financial compensation. Sixty-two (19%) reported offering financial compensation to all patient partners, 17 (5%) studies reported offering financial compensation to some patient partners, 32 (10%) explicitly reported that patient partners were not offered any form of financial compensation, and 205 studies (65%) did not report on financial compensation (Table 4).

**Table 4:** Financial compensation details (n = 316)

Financial compensation details	Number of studies (%)
*Did the authors offer financial compensation to patient partners?*	
Yes, financial compensation was offered to all patient partners	62 (19)
Financial compensation was offered to some patient partners	17 (5)
No, patient partners were not offered financial compensation	32 (10)
Not reported	205 (65)
*Type of financial compensation offered to patient partners (n = 79)*	
Honoraria or stipend	47 (59)
Salary	4 (5)
Gift card (e.g., fee, voucher)	13 (16)
Scholarship	1 (1)
Not reported	20 (26)
*Amount (rate)	
$12–997 per task completed (i.e. attended meeting, review document, video feature)	15 (19)
$31–94 per half day meeting	4 (5)
$12–42 per hour	7 (9)
0.1 full-time equivalent for the duration of the project	1 (1)
Not reported	57 (73)
*Amount (total)	
$15–77	3 (4)
$77–153	5 (6)
$376	1 (1)
Sufficient value to take a friend for dinner	1 (1)
Not reported	68 (87)
*Payment frequency*	
Annually	1 (1)
One payment	13 (17)
Paid at each meeting	4 (5)
Not reported	61 (77)

*Currency presented in USD (conversions were made based on September 2022 rates)

We identified several reported methods of offering financial compensation including honoraria (e.g., cheques, stipend) (58%, n = 46), gift cards (16%, n = 13), salary (5%, n = 4) and scholarship (1%, n = 1). Sixteen (20%) studies reported offering task-based financial compensation (e.g., attended meeting, document review), while other studies used units of time (e.g., half-day meeting, per hour, full-time-equivalent salary rate) to inform the monetary value of financial compensation (14%, n = 11). Reported compensation rates ranged from $12 to $42 USD per hour or $31 to $94 USD for attendance at a half-day meeting (which at approximately 4 h would convert to $7.75–23.50 USD per hour) (Table 4). However, the majority of studies in which financial compensation was provided did not report the monetary value of financial compensation (72%, n = 57). In terms of payment frequency, thirteen (16%) studies reported providing financial compensation as a one-time payment, four (5%) studies paid patient partners immediately after completion of a task, and one (1%) study provided patient partners with an annual payment [38]. Most studies (77%, n = 61) did not report on payment frequency.

The most referenced documents used to inform financial compensation strategies were developed by the NIHR and INVOLVE (n = 22) including *Payment and Recognition for Public Involvement* [15]*, Policy on payment of fees and expenses for members of the public actively involved with Involve* [39]*,* and *Recognition payments for public contributors* [40]*.* Five studies used local minimum wage rates or national costs of living to inform the monetary value of financial compensation offered to patient partners.

### Subgroup analysis

Studies that reported receipt of funding were significantly more likely to report financial compensation of patient partners when compared to studies that did not receive funding or did not report funding (${\chi }^{2}$=6.614, p < 0.01).Additionally, there was a significant difference in reporting patient partner financial compensation between studies that engaged patients at different levels (*Consult, Collaborate* and *Empower*) (${\chi }^{2}$= 42.41, p < 0.01). This finding suggests that reporting patient partner financial compensation and the level at which patients were engaged, are not independent of each other. Indeed, 42% (n = 47) of studies that achieved *Consult* as the highest level of engagement (n = 111) reported offering financial compensation to all or some patient partners, compared to 67% (n = 49) of studies that achieved *Collaborate* as the highest level of engagement (n = 73) and 95% (n = 18) of studies that achieved *Empower* as the highest level of engagement (n = 19).

A post-hoc subgroup analysis of studies where the corresponding author was based in Canada, United Kingdom and United States compared to studies where corresponding authors were based elsewhere found no significant difference in reporting patient partner financial compensation (${\chi }^{2}$=0.669, p = 0.41).

### Benefits, challenges, barriers and enablers of financial compensation

A total of 32 studies (10%) reported benefits, challenges, barriers or enablers of financial compensation. The most common themes indicating benefits of financially compensating patient partners were “support patient partner participation” and “facilitate long term engagement across the research project”, since offering financial compensation can enable patient partners to allocate more time to the research project (e.g., attending more team meetings) (Table 5). One identified theme indicating a challenge to patient partner financial compensation was “financial payments can jeopardize disability or social security payments or impact income tax rates” since financial compensation can impact other income sources. Two themes indicating common barriers to offering financial compensation were “budget constraints” or “lack of funding for the study” and “lack of an institutional policy or guidance document” to inform compensation strategies.

**Table 5:** Reported benefits, challenges, barriers and enablers of patient partner financial compensation

Themes	Number of studies
*Benefits (n = 16)*	
Facilitates patient partner participation in research activities across the research project and supports long term engagement	7
Tangible method to demonstrate patient partner appreciation and	5
Supports a sense of equality among team members	4
*Challenges (n = 3)*	
Financial payments can jeopardize disability or social security payments or impact income tax rates (United Kingdom, Netherlands)	3
*Barriers (n = 14)*	
Budget constraints or the research project did not receive funding	6
Lack of an institutional policy or guidance document	6
Failing to consider the cost of patient engagement at the study design or funding application phase	2
*Enablers (n = 9)*	
Sufficient resources and funding	6
Institutional and financial support at the pre-funding stage to support engagement at project onset	3

## Discussion

We conducted this systematic review to enhance our understanding of how patient partner recognition and compensation practices are reported, with the aim of providing valuable support to the research community in making informed decisions and formulating policies regarding patient compensation. We found that, among studies citing the GRIPP or GRIPP2 reporting guidelines, the majority of studies involving patients in research report non-financial compensation, while only a minority report financial compensation for patients.

The most common reported methods of non-financial compensation were informal acknowledgement and co-authorship. The level of reported co-authorship was much higher than levels reported in the literature, and was consistent with the highest levels of co-authorship reported in this journal [41]. Indeed, a review of systematic reviews published between 2011 and 2020 identified only 37 reviews that included a patient partner co-author [42]. Our results are, however, in line with previous research that found that acknowledgement was more common than co-authorship, even within the field of participatory research [43]. Although non-financial compensation (e.g. authorship or acknowledgements) was reported more frequently than financial compensation, we would also note drawbacks and potential threats to this approach. For instance, while there are academic career benefits from co-authorship for research team members, patient partners engage in research for different reasons and may not see the benefits or disadvantages of authorship in the same way. In some instances, co-authorship may even be refused by patient partners with lived experience of a stigmatized condition [44].

In terms of financial compensation, we identified substantial variation in the monetary value assigned, with reported compensation rates ranging from $12 to $42 USD per hour and $31 to $94 USD for attendance at a half-day meeting. We also found an association between the reported level of engagement (e.g*., Consult, Collaborate,* or *Empower*) and the reporting of financial compensation [35]. While we were unable to examine the extent to which the level of engagement was associated with the compensation rates reported, this would be consistent with recommendations from various compensation guidance documents that suggest different compensation rates based on the roles of patient partners in the research project [16, 18, 45–47].

Among the studies that did report financial compensation, researchers identified two key benefits: fostering long-term patient engagement in research and serving as a tangible means to express appreciation for patient partners' contributions. Moreover, barriers reported included “budget constraints” or “lack of funding for the study”. This is consistent with our subgroup analysis findings that suggest that studies reporting receipt of funding were significantly more likely to report financial compensation of patient partners. Notably, 12% of studies that received funding explicitly reported funding to support patient engagement.

While offering financial compensation to patient partners can have a positive impact on their engagement and is encouraged by most patient-oriented organizations, our review also identified potential barriers and challenges in implementing such compensation. A key barrier we identified is the lack of institutional policies and guidance, which limits research teams' ability to offer financial compensation to patient partners. This finding aligns with the barriers identified by individuals who have experience as patient partners in research. In their work, Richards et al. emphasized the crucial role of institutions in supporting patient partner compensation [48], particularly in developing or modifying existing contractual agreements to accommodate patient partnerships and logistics of processing payments. Therefore, it is important for institutions to adopt compensation guidelines and establish effective payment procedures, which can assist researchers in offering financial compensation to patient partners.

When developing a compensation strategy it is important to consider potential threats of financial compensation. Indeed, two studies in our review reported that they deliberately did not offer financial compensation to ensure patient partners were free to express their thoughts without any pressures associated with receiving payments. Keeping financial compensation at a level that reflects appreciation and is approved by patient partners may address this challenge. We also identified “*jeopardization of disability or social security payments”* as a significant risk of offering financial compensation, which further highlights the importance of considering patient partner preferences and circumstances. By carefully considering these benefits and threats of financial and non-financial compensation, researchers and patient partners can co-develop a compensation strategy that appropriately balances recognition, respect for autonomy, and potential threats associated with financial compensation.

Finally, we would suggest that it is imperative that researchers are transparent in reporting all aspects of their health research [49, 50] and reporting important details of patient engagement in research should be treated as no less important. Determining the essential patient engagement compensation elements to report, including financial aspects, requires further evaluation to incentivize reporting of patient partner compensation.

### Limitations of the study

Our systematic review has certain limitations that should be acknowledged. First, our search strategy is limited to studies that cited the GRIPP reporting checklists, thus published patient engagement research that did not use the GRIPP checklists were not included. As stated above, it would not be feasible to conduct a broad literature search given the paucity of reporting patient engagement in health research [27]. Thus, it is crucial to consider that reporting of compensation practices may be different in studies that did not cite the GRIPP checklist. Second, the majority of included studies were written by researchers, which may introduce bias towards the researcher perspective. It is important to consider the experiences of patient partners themselves to better understand benefits, challenges, barriers and enablers to financial and non-financial compensation.

## Conclusions

Our systematic review contributes to an area of patient engagement research where very little evidence exists. We found that non-financial compensation was more commonly reported than financial. Importantly, the details of financial compensation were rarely reported and highly variable although we did observe a signal that an increased level of engagement was associated with offering financial compensation. Our findings also suggest that adequate funding and budget guidance may support researchers in offering financial compensation to patient partners. Our work supports the need for the research community to better report patient engagement activities including patient compensation practices.

## Data Availability

The datasets supporting the conclusions of this article are included within the article and its additional files.

## Appendix 1: PRISMA checklist

**Table:**

Section and Topic	Item #	Checklist item	Location where item is reported
*Title*			
Title	1	Identify the report as a systematic review	1
*Abstract*			
Abstract	2	See the PRISMA 2020 for Abstracts checklist	3
*Introduction*			
Rationale	3	Describe the rationale for the review in the context of existing knowledge	4
Objectives	4	Provide an explicit statement of the objective(s) or question(s) the review addresses	4–5
*Methods*			
Eligibility criteria	5	Specify the inclusion and exclusion criteria for the review and how studies were grouped for the syntheses	5, 6, 29
Information sources	6	Specify all databases, registers, websites, organisations, reference lists and other sources searched or consulted to identify studies. Specify the date when each source was last searched or consulted	6
Search strategy	7	Present the full search strategies for all databases, registers and websites, including any filters and limits used	6
Selection process	8	Specify the methods used to decide whether a study met the inclusion criteria of the review, including how many reviewers screened each record and each report retrieved, whether they worked independently, and if applicable, details of automation tools used in the process	6
Data collection process	9	Specify the methods used to collect data from reports, including how many reviewers collected data from each report, whether they worked independently, any processes for obtaining or confirming data from study investigators, and if applicable, details of automation tools used in the process	7
Data items	10a	List and define all outcomes for which data were sought. Specify whether all results that were compatible with each outcome domain in each study were sought (e.g. for all measures, time points, analyses), and if not, the methods used to decide which results to collect	7, 30
	10b	List and define all other variables for which data were sought (e.g. participant and intervention characteristics, funding sources). Describe any assumptions made about any missing or unclear information	7, 30
Study risk of bias assessment	11	Specify the methods used to assess risk of bias in the included studies, including details of the tool(s) used, how many reviewers assessed each study and whether they worked independently, and if applicable, details of automation tools used in the process	7
Effect measures	12	Specify for each outcome the effect measure(s) (e.g. risk ratio, mean difference) used in the synthesis or presentation of results	N/A
Synthesis methods	13a	Describe the processes used to decide which studies were eligible for each synthesis (e.g. tabulating the study intervention characteristics and comparing against the planned groups for each synthesis (item #5))	7, 8
	13b	Describe any methods required to prepare the data for presentation or synthesis, such as handling of missing summary statistics, or data conversions	7,8
	13c	Describe any methods used to tabulate or visually display results of individual studies and syntheses	7, 8
	13d	Describe any methods used to synthesize results and provide a rationale for the choice(s). If meta-analysis was performed, describe the model(s), method(s) to identify the presence and extent of statistical heterogeneity, and software package(s) used	7, 8
	13e	Describe any methods used to explore possible causes of heterogeneity among study results (e.g. subgroup analysis, meta-regression)	N/A
	13f	Describe any sensitivity analyses conducted to assess robustness of the synthesized results	N/A
Reporting bias assessment	14	Describe any methods used to assess risk of bias due to missing results in a synthesis (arising from reporting biases)	N/A
Certainty assessment	15	Describe any methods used to assess certainty (or confidence) in the body of evidence for an outcome	N/A
*Results*			
Study selection	16a	Describe the results of the search and selection process, from the number of records identified in the search to the number of studies included in the review, ideally using a flow diagram	9
	16b	Cite studies that might appear to meet the inclusion criteria, but which were excluded, and explain why they were excluded	9, 31–47
Study characteristics	17	Cite each included study and present its characteristics	9, 31–47
Risk of bias in studies	18	Present assessments of risk of bias for each included study	N/A
Results of individual studies	19	For all outcomes, present, for each study: (a) summary statistics for each group (where appropriate) and (b) an effect estimate and its precision (e.g. confidence/credible interval), ideally using structured tables or plots	9–12, 19–23
Results of syntheses	20a	For each synthesis, briefly summarise the characteristics and risk of bias among contributing studies	9–12, 19–23
	20b	Present results of all statistical syntheses conducted. If meta-analysis was done, present for each the summary estimate and its precision (e.g. confidence/credible interval) and measures of statistical heterogeneity. If comparing groups, describe the direction of the effect	9–12, 19–23
	20c	Present results of all investigations of possible causes of heterogeneity among study results	N/A
	20d	Present results of all sensitivity analyses conducted to assess the robustness of the synthesized results	N/A
Reporting biases	21	Present assessments of risk of bias due to missing results (arising from reporting biases) for each synthesis assessed	N/A
Certainty of evidence	22	Present assessments of certainty (or confidence) in the body of evidence for each outcome assessed	N/A
*Discussion*			
Discussion	23a	Provide a general interpretation of the results in the context of other evidence	12–13
	23b	Discuss any limitations of the evidence included in the review	13
	23c	Discuss any limitations of the review processes used	13
	23d	Discuss implications of the results for practice, policy, and future research	14–15
*Other information*			
Registration and protocol	24a	Provide registration information for the review, including register name and registration number, or state that the review was not registered	5
	24b	Indicate where the review protocol can be accessed, or state that a protocol was not prepared	5
	24c	Describe and explain any amendments to information provided at registration or in the protocol	N/A
Support	25	Describe sources of financial or non-financial support for the review, and the role of the funders or sponsors in the review	14
Competing interests	26	Declare any competing interests of review authors	14
Availability of data, code and other materials	27	Report which of the following are publicly available and where they can be found: template data collection forms; data extracted from included studies; data used for all analyses; analytic code; any other materials used in the review	14

## Appendix 2: GRIPP II short form reporting checklist

**Table:**

Section and topic	Item
1: Aim	Conduct a systematic review to assess the current landscape of reporting patient partner financial compensation and identifying current compensation practices. To partner with a patient partner throughout the development and conduct of the systematic review
2: Methods	One patient partner (MS) was recruited to join the research team through personal referral. MS was involved in developing the protocol, defining compensation terms, identifying data items for extraction, analyzing systematic review results and contributed to edits of this paper. MS attended virtual team meetings and continued to meet with GF monthly. MS was offered financial compensation and co-authorship in recognition of her contributions to the research project
3: Results	Patient engagement contributed to the study in several ways including: Informing the project proposal with the patient partner experience: MS is well integrated in the patient engagement field and has a wealth of experience being a patient partner for several organizations. MS has experience with various methods of compensation, barriers to financial compensation and the different perspectives that patient partners have on financial compensation Co-developed definitions for non-financial and financial compensation Categorizing methods of compensation as reimbursement, financial or non-financial compensation Identified opportunities to present the systematic review proposal and findings to larger panels of patient partners
4: Discussion	Overall, patient engagement was successful in informing review development and conduct. Additionally, the research team learned a lot about the patient partner experience with financial compensation and institutions are recognizing patient partners for their expertise through discussions with MS about her unique experiences. It was helpful that MS was familiar with most team members before joining the research team and that members of the team had experience with patient engagement The systematic review was conducted within a year. At the beginning of the project, we co-developed a timeline and budget to reflect the number of hours that MS devoted to the project. In the future, we will refer back to this timeline at the mid-term mark to ensure that the number of hours budgeted for were accurate
5: Reflections	Engagement was embedded within the research project and MS was a member of the research team. MS connected the research team with groups of patient partners who were interested in the systematic review findings. In turn, these connections yielded opportunities to connect with patient groups and disseminate review findings to an important stakeholder group. This would not have been possible without MS

## Appendix 3: Data extraction items

**Table:**

Data item	
1. Corresponding author name, e-mail address, country of residence, and institutional affiliation at time of publication	
2. Publication title	
3. Year of publication	
4. Journal/Source of publication	
5. Funding details (e.g. source of funding, whether funding was received specifically to support patient engagement)	
6. Type of stakeholder engaged (e.g. patients, caregivers, community member etc.)	
7. Number of patient partners engaged	
8. Length of engagement (i.e. whether patient partners were engaged once or multiple times throughout the project)	
9. Research element where patient partners contributed (e.g. funding, priority-setting, governance, study design, data collection, data analysis, dissemination, ethics approval etc.) [2]	
10. Level of patient partner engagement (as defined by Involve (33))	
11. Non-financial compensation offered to patient partners (e.g. co-authorship, gifts, refreshments etc.)	
12. Did authors report on offering financial compensation to patient partners (patient partners need not accept)? (Yes or No)	
13. Where are details of financial compensation reported in the manuscript? (e.g. methods, results, discussion)	
(a) Type of financial compensation (e.g. honoraria, salary, cash etc.)	
(b) Amount (rate and total)	
(c) Payment frequency (e.g. bi-weekly, one-payment etc.)	
(d) Reported guidelines or policies used to guide financial compensation	
14. Stated reason for financially compensating patient partners or stated reason for not financially compensating patient partners	
15. Reported benefits and challenges to financially compensating patient partners	
16. Reported barriers and enablers to financially compensating patient partners	

## Appendix 4: Included studies (n = 316)

1. Zoellner, J. M. *et al.* Advancing engagement and capacity for rural cancer control: a mixed-methods case study of a Community-Academic Advisory Board in the Appalachia region of Southwest Virginia. *Research Involvement and Engagement* 7, (2021).
2. Yu, R., Hanley, B., Denegri, S., Ahmed, J. & McNally, N. J. Evaluation of a patient and public involvement training programme for researchers at a large biomedical research centre in the UK. *BMJ Open* 11, (2021).
3. Young, R. *et al.* Using nominal group technique to advance power assisted exercise equipment for people with stroke. *Research Involvement and Engagement* 7, (2021).
4. Young, A. *et al.* The lived experience and legacy of pragmatics for deaf and hard of hearing children. *Pediatrics* 146, (2020).
5. Woodford, J., Farrand, P., Hagström, J., Hedenmalm, L. & von Essen, L. Internet-administered cognitive behavioral therapy for common mental health difficulties in parents of children treated for cancer: Intervention development and description study. *JMIR Formative Research* 5, (2021).
6. Williams, M. A. *et al.* Active treatment for idiopathic adolescent scoliosis (ACTIvATeS): A feasibility study. *Health Technology Assessment* 19, (2015).
7. White, K. *et al.* Chronic Headache Education and Self-management Study (CHESS) - A mixed method feasibility study to inform the design of a randomised controlled trial. *BMC Medical Research Methodology* 19, (2019).
8. Warner, G., Baghdasaryan, Z., Osman, F., Lampa, E. & Sarkadi, A. ‘I felt like a human being’—An exploratory, multi-method study of refugee involvement in the development of mental health intervention research. *Health Expectations* 24, 30–39 (2021).
9. von Scheven, E., Nahal, B. K., Cohen, I. C., Kelekian, R. & Franck, L. S. Research Questions that Matter to Us: priorities of young people with chronic illnesses and their caregivers. *Pediatric Research* 89, 1659–1663 (2021).
10. Vogsen, M., Geneser, S., Rasmussen, M. L., Hørder, M. & Hildebrandt, M. G. Learning from patient involvement in a clinical study analyzing PET/CT in women with advanced breast cancer. *Research Involvement and Engagement* 6, (2020).
11. Vat, L. E. *et al.* Giving patients a voice: A participatory evaluation of patient engagement in Newfoundland and Labrador Health Research. *Research Involvement and Engagement* 6, (2020).
12. Vanderhout, S. M. *et al.* Patient and family engagement in the development of core outcome sets for two rare chronic diseases in children. *Research Involvement and Engagement* 7, (2021).
13. Van Schelven, F., Van Der Meulen, E., Kroeze, N., Ketelaar, M. & Boeije, H. Patient and public involvement of young people with a chronic condition: Lessons learned and practical tips from a large participatory program. *Research Involvement and Engagement* 6, (2020).
14. van Rooijen, M. *et al.* How to foster successful implementation of a patient reported experience measurement in the disability sector: an example of developing strategies in co-creation. *Research Involvement and Engagement* 7, (2021).
15. van Beinum, A., Talbot, H., Hornby, L., Fortin, M. C. & Dhanani, S. Engaging family partners in deceased organ donation research—a reflection on one team’s experience. *Canadian Journal of Anesthesia* 66, 406–413 (2019).
16. Udayaraj, U. P. *et al.* Establishing a tele-clinic service for kidney transplant recipients through a patient-codesigned quality improvement project. *BMJ Open Quality* 8, (2019).
17. Turner, G., Aiyegbusi, O. L., Price, G., Skrybant, M. & Calvert, M. Moving beyond project-specific patient and public involvement in research. *Journal of the Royal Society of Medicine* 113, 16–23 (2020).
18. Tsang, V. W. L., Fletcher, S., Thompson, C. & Smith, S. A novel way to engage youth in research: evaluation of a participatory health research project by the international children’s advisory network youth council. *International Journal of Adolescence and Youth* 25, 676–686 (2020).
19. Troya, M. I. *et al.* Patient and Public Involvement and Engagement in a doctoral research project exploring self-harm in older adults. *Health Expectations* 22, 617–631 (2019).
20. Treiman, K. *et al.* Engaging Patient Advocates and Other Stakeholders to Design Measures of Patient-Centered Communication in Cancer Care. *Patient* 10, 93–103 (2017).
21. Tomlinson, J., Medlinskiene, K., Cheong, V. L., Khan, S. & Fylan, B. Patient and public involvement in designing and conducting doctoral research: The whys and the hows. *Research Involvement and Engagement* 5, (2019).
22. Toledo-Chávarri, A. *et al.* Co-design process of a virtual community of practice for the empowerment of people with ischemic heart disease. *International Journal of Integrated Care* 20, 1–13 (2020).
23. Toledo-Chávarri, A. *et al.* Toward a Strategy to Involve Patients in Health Technology Assessment in Spain. *International Journal of Technology Assessment in Health Care* 35, 92–98 (2019).
24. Tierney, E. *et al.* A critical analysis of the implementation of service user involvement in primary care research and health service development using normalization process theory. *Health Expectations* 19, 501–515 (2016).
25. Thomas, M. *et al.* Patient preferences to value health outcomes in rheumatology clinical trials: Report from the OMERACT special interest group✰. *Seminars in Arthritis and Rheumatism* 51, 919–924 (2021).
26. Thomas, K. S. *et al.* Randomised controlled trial of silk therapeutic garments for the management of atopic eczema in children: The CLOTHES trial. *Health Technology Assessment* 21, 1–259 (2017).
27. Tchajkova, N., Ethans, K. & Smith, S. D. Inside the lived perspective of life after spinal cord injury: a qualitative study of the desire to live and not live, including with assisted dying. *Spinal Cord* 59, 485–492 (2021).
28. Taylor, J. *et al.* Making the patient voice heard in a research consortium: experiences from an EU project (IMI-APPROACH). *Research Involvement and Engagement* 7, (2021).
29. Tallantyre, E. C. *et al.* Achieving effective patient and public involvement in international clinical trials in neurology. *NEUROLOGY-CLINICAL PRACTICE* 10, 265–272.
30. Synnot, A. J. *et al.* Consumer engagement critical to success in an Australian research project: Reflections from those involved. *Australian Journal of Primary Health* 24, 197–203 (2018).
31. Swaithes, L. *et al.* Understanding the uptake of a clinical innovation for osteoarthritis in primary care: a qualitative study of knowledge mobilisation using the i-PARIHS framework. *Implementation Science* 15, (2020).
32. Stocker, R., Brittain, K., Spilsbury, K. & Hanratty, B. Patient and public involvement in care home research: Reflections on the how and why of involving patient and public involvement partners in qualitative data analysis and interpretation. *Health Expectations* 24, 1349–1356 (2021).
33. Staniszewska, S. *et al.* Developing a Framework for Public Involvement in Mathematical and Economic Modelling: Bringing New Dynamism to Vaccination Policy Recommendations. *Patient* 14, 435–445 (2021).
34. Staniszewska, S., Denegri, S., Matthews, R. & Minogue, V. Reviewing progress in public involvement in NIHR research: Developing and implementing a new vision for the future. *BMJ Open* 8, (2018).
35. Staniszewska, S. *et al.* GRIPP2 reporting checklists: Tools to improve reporting of patient and public involvement in research. *Research Involvement and Engagement* 3, (2017).
36. Staniszewska, S. *et al.* Developing the evidence base of patient and public involvement in health and social care research: The case for measuring impact. *International Journal of Consumer Studies* 35, 628–632 (2011).
37. Staats, K., Grov, E. K., Husebø, B. & Tranvåg, O. Framework for patient and informal caregiver participation in research (PAICPAIR): Part 1. *Advances in Nursing Science* 43, E58–E70 (2020).
38. Sohy, D. *et al.* Outside in–inside out. Creating focus on the patient–a vaccine company perspective. *Human Vaccines and Immunotherapeutics* 14, 1509–1514 (2018).
39. Snooks, H. A. *et al.* What are emergency ambulance services doing to meet the needs of people who call frequently? A national survey of current practice in the United Kingdom. *BMC Emergency Medicine* 19, (2019).
40. Snape, D. *et al.* Exploring areas of consensus and conflict around values underpinning public involvement in health and social care research: A modified Delphi study. *BMJ Open* 4, (2014).
41. Snape, D. *et al.* Exploring perceived barriers, drivers, impacts and the need for evaluation of public involvement in health and social care research: A modified Delphi study. *BMJ Open* 4, (2014).
42. Smits, D. W., Van Meeteren, K., Klem, M., Alsem, M. & Ketelaar, M. Designing a tool to support patient and public involvement in research projects: The Involvement Matrix. *Research Involvement and Engagement* 6, (2020).
43. Smith, H. *et al.* Defining and evaluating novel procedures for involving patients in core outcome set research: Creating a meaningful long list of candidate outcome domains. *Research Involvement and Engagement* 4, (2018).
44. Smith, A. B. *et al.* Patient and public involvement in the design and conduct of a large, pragmatic observational trial to investigate recurrent, high-risk non–muscle-invasive bladder cancer. *Cancer* (2021).
45. Slåtsveen, R. E., Wibe, T., Halvorsrud, L. & Lund, A. Needs-led research: a way of employing user involvement when devising research questions on the trust model in community home-based health care services in Norway. *Research Involvement and Engagement* 7, (2021).
46. Skovlund, P. C. *et al.* The impact of patient involvement in research: a case study of the planning, conduct and dissemination of a clinical, controlled trial. *Research Involvement and Engagement* 6, (2020).
47. Skivington, K. *et al.* Development of the framework. *HEALTH TECHNOLOGY ASSESSMENT* 25, 1-+.
48. Shields, G. E., Brown, L., Wells, A., Capobianco, L. & Vass, C. Utilising Patient and Public Involvement in Stated Preference Research in Health: Learning from the Existing Literature and a Case Study. *Patient* 14, 399–412 (2021).
49. Shaw, L. *et al.* An extended stroke rehabilitation service for people who have had a stroke: the EXTRAS RCT. *HEALTH TECHNOLOGY ASSESSMENT* 24, 1-+.
50. Sellars, E., Pavarini, G., Michelson, D., Creswell, C. & Fazel, M. Young people’s advisory groups in health research: Scoping review and mapping of practices. *Archives of Disease in Childhood* 106, 698–704 (2021).
51. Seljelid, B. *et al.* Content and system development of a digital patient-provider communication tool to support shared decision making in chronic health care: InvolveMe. *BMC Medical Informatics and Decision Making* 20, (2020).
52. Sehlbach, C. *et al.* Perceptions of people with respiratory problems on physician performance evaluation-A qualitative study. *HEALTH EXPECTATIONS* 23, 247–255.
53. Seeralan, T. *et al.* Patient involvement in developing a patient-targeted feedback intervention after depression screening in primary care within the randomized controlled trial GET.FEEDBACK.GP. *Health Expectations* 24, 95–112 (2021).
54. Schulte, F. S. M., Chalifour, K., Eaton, G. & Garland, S. N. Quality of life among survivors of adolescent and young adult cancer in Canada: A Young Adults With Cancer in Their Prime (YACPRIME) study. *Cancer* 127, 1325–1333 (2021).
55. Scholz, B. & Bevan, A. Toward more mindful reporting of patient and public involvement in healthcare. *Research Involvement and Engagement* 7, (2021).
56. Schmidt, A. M., Schiøttz-Christensen, B., Foster, N. E., Laurberg, T. B. & Maribo, T. The effect of an integrated multidisciplinary rehabilitation programme alternating inpatient interventions with home-based activities for patients with chronic low back pain: a randomized controlled trial. *Clinical Rehabilitation* 34, 382–393 (2020).
57. Schilling, I. *et al.* Patients’ and researchers’ experiences with a patient board for a clinical trial on urinary tract infections. *Research Involvement and Engagement* 5, (2019).
58. Sass, R. *et al.* Patient, Caregiver, and Provider Perspectives on Challenges and Solutions to Individualization of Care in Hemodialysis: A Qualitative Study. *Canadian Journal of Kidney Health and Disease* 7, (2020).
59. Santer, M. *et al.* Adding emollient bath additives to standard eczema management for children with eczema: The BATHE RCT. *Health Technology Assessment* 22, 1–116 (2018).
60. Sangill, C., Buus, N., Hybholt, L. & Berring, L. L. Service user’s actual involvement in mental health research practices: A scoping review. *International Journal of Mental Health Nursing* 28, 798–815 (2019).
61. Sale, J. E. M. *et al.* Perspectives of patients with depression and chronic pain about bone health after a fragility fracture: A qualitative study. *Health Expectations* (2021).
62. Sabey, A. & Hardy, H. Views of newly-qualified GPs about their training and preparedness: Lessons for extended generalist training. *British Journal of General Practice* 65, e270–e277 (2015).
63. Sabey, A. & Hardy, H. Prepared for commissioning? A qualitative study into the views of recently qualified GPs. *Education for Primary Care* 24, 314–320 (2013).
64. Rushton, A. *et al.* Patient journey following lumbar spinal fusion surgery (FuJourn): A multicentre exploration of the immediate post-operative period using qualitative patient diaries. *PLoS ONE* 15, (2020).
65. Romsland, G. I., Milosavljevic, K. L. & Andreassen, T. A. Facilitating non-tokenistic user involvement in research. *Research Involvement and Engagement* 5, (2019).
66. Rodgers, H. *et al.* Robot-assisted training compared with an enhanced upper limb therapy programme and with usual care for upper limb functional limitation after stroke: The ratuls three-group RCT. *Health Technology Assessment* 24, 1–232 (2020).
67. Roche, P. *et al.* Valuing All Voices: refining a trauma-informed, intersectional and critical reflexive framework for patient engagement in health research using a qualitative descriptive approach. *Research Involvement and Engagement* 6, (2020).
68. Robler, S. K. *et al.* Hearing Norton Sound: community involvement in the design of a mixed methods community randomized trial in 15 Alaska Native communities. *Research Involvement and Engagement* 6, (2020).
69. Richards, D. P. *et al.* Guidance on authorship with and acknowledgement of patient partners in patient-oriented research. *Research Involvement and Engagement* 6, (2020).
70. Retzer, A. *et al.* Development of a core outcome set for use in community-based bipolar trials—A qualitative study and modified Delphi. *PLoS ONE* 15, (2020).
71. Racine, E. *et al.* 'It just wasn’t going to be heard’: A mixed methods study to compare different ways of involving people with diabetes and health-care professionals in health intervention research. *Health Expectations* 23, 870–883 (2020).
72. Puggaard, R. S. *et al.* Establishing research priorities related to osteoarthritis care via stakeholder input from patients. *Danish Medical Journal* 68, 1–8 (2021).
73. Price, J., Rushton, A., Tyros, V. & Heneghan, N. R. Expert consensus on the important chronic non-specific neck pain motor control and segmental exercise and dosage variables: An international e-Delphi study. *PLoS ONE* 16, (2021).
74. Price, A. *et al.* SMOOTH: Self-Management of Open Online Trials in Health analysis found improvements were needed for reporting methods of internet-based trials. *Journal of Clinical Epidemiology* 105, 27–39 (2019).
75. Price, A. *et al.* Frequency of reporting on patient and public involvement (PPI) in research studies published in a general medical journal: A descriptive study. *BMJ Open* 8, (2018).
76. Price, A. *et al.* Mind the gap in clinical trials: A participatory action analysis with citizen collaborators. *Journal of Evaluation in Clinical Practice* 23, 178–184 (2017).
77. Price, A. *et al.* Patient and Public Involvement in research: A journey to co-production. *Patient Education and Counseling* (2021).
78. Price, A. *et al.* Patient and public involvement in the design of clinical trials: An overview of systematic reviews. *Journal of Evaluation in Clinical Practice* 24, 240–253 (2018).
79. Poolman, M. *et al.* Carer administration of as-needed subcutaneous medication for breakthrough symptoms in people dying at home: The cariad feasibility rct. *Health Technology Assessment* 24, 1–149 (2020).
80. Pomey, M. P. *et al.* Assessing and promoting partnership between patients and health-care professionals: Co-construction of the CADICEE tool for patients and their relatives. *HEALTH EXPECTATIONS* 24, 1230–1241.
81. Pollock, A. *et al.* Development of the ACTIVE framework to describe stakeholder involvement in systematic reviews. *Journal of Health Services Research and Policy* 24, 245–255 (2019).
82. Pollock, A. *et al.* Stakeholder involvement in systematic reviews: A scoping review. *Systematic Reviews* 7, (2018).
83. Poland, F., Charlesworth, G., Leung, P. & Birt, L. Embedding patient and public involvement: Managing tacit and explicit expectations. *Health Expectations* 22, 1231–1239 (2019).
84. Poitras, M. E. *et al.* Decisional needs assessment of patients with complex care needs in primary care. *Journal of Evaluation in Clinical Practice* 26, 489–502 (2020).
85. Ploetner, C. *et al.* Understanding and improving the experience of claiming social security for mental health problems in the west of Scotland: A participatory social welfare study. *Journal of Community Psychology* 48, 675–692 (2020).
86. Pezaro, S., Pearce, G. & Bailey, E. Childbearing women’s experiences of midwives’ workplace distress: Patient and public involvement. *British Journal of Midwifery* 26, 659–669 (2018).
87. Patchwood, E. *et al.* Organising Support for Carers of Stroke Survivors (OSCARSS): a cluster randomised controlled trial with economic evaluation. *BMJ Open* 11, (2021).
88. Paskins, Z. *et al.* Quality and effectiveness of osteoporosis treatment decision aids: a systematic review and environmental scan. *OSTEOPOROSIS INTERNATIONAL* 31, 1837–1851.
89. Parslow, R. M. *et al.* Development of a conceptual framework to underpin a health-related quality of life outcome measure in paediatric chronic fatigue syndrome/myalgic encephalopathy (CFS/ME): prioritisation through card ranking. *Quality of Life Research* 29, 1169–1181 (2020).
90. Parr, J. *et al.* Parent-delivered interventions used at home to improve eating, drinking and swallowing in children with neurodisability: The feeds mixed-methods study. *Health Technology Assessment* 25, 1–208 (2021).
91. Parkash, V. *et al.* Assessing public perception of a sand fly biting study on the pathway to a controlled human infection model for cutaneous leishmaniasis. *Research Involvement and Engagement* 7, (2021).
92. Pandya-Wood, R., Barron, D. S. & Elliott, J. A framework for public involvement at the design stage of NHS health and social care research: Time to develop ethically conscious standards. *Research Involvement and Engagement* 3, (2017).
93. Østerås, N. *et al.* Improving osteoarthritis management in primary healthcare: results from a quasi-experimental study. *BMC Musculoskeletal Disorders* 22, (2021).
94. Ogourtsova, T. *et al.* Patient engagement in an online coaching intervention for parents of children with suspected developmental delays. *Developmental Medicine and Child Neurology* 63, 668–674 (2021).
95. Odgers, H. L. *et al.* Research priority setting in childhood chronic disease: A systematic review. *Archives of Disease in Childhood* 103, 942–951 (2018).
96. O’Regan, M. *et al.* Public & patient involvement to guide research in wound care in an Irish context. A round table report. *Journal of Tissue Viability* 29, 7–11 (2020).
97. Nunn, J. S. *et al.* Involving elderly research participants in the co-design of a future multi-generational cohort study. *Research Involvement and Engagement* 7, (2021).
98. Nunn, J. S., Gwynne, K., Gray, S. & Lacaze, P. Involving people affected by a rare condition in shaping future genomic research. *Research Involvement and Engagement* 7, (2021).
99. Noblet, T. *et al.* Independent prescribing by advanced physiotherapists for patients with low back pain in primary care: A feasibility trial with an embedded qualitative component. *PLOS ONE* 15,.
100. Nissen, E. R. *et al.* Patient involvement in the development of a psychosocial cancer rehabilitation intervention: Evaluation of a shared working group with patients and researchers. *Research Involvement and Engagement* 4, (2018).
101. Ní Shé, É. *et al.* Minding the gap: Identifying values to enable public and patient involvement at the pre-commencement stage of research projects. *Research Involvement and Engagement* 6, (2020).
102. Newburn, M., Scanlon, M., Plachcinski, R. & Macfarlane, A. J. Involving service users in the Birth Timing project, a data linkage study analysing the timing of births and their outcomes. *International Journal of Population Data Science* 5, (2020).
103. Muir, R. *et al.* Patient involvement in surgical wound care research: A scoping review. *International Wound Journal* 17, 1462–1482 (2020).
104. Mrklas, K. J. *et al.* Co-design in the development of a mobile health app for the management of knee osteoarthritis by patients and physicians: Qualitative study. *JMIR mHealth and uHealth* 8, (2020).
105. Moses, C., Flegg, K. & Dimaras, H. Patient knowledge, experiences and preferences regarding retinoblastoma and research: A qualitative study. *Health Expectations* 23, 632–643 (2020).
106. Morris, R. L. *et al.* Developing a patient safety guide for primary care: A co-design approach involving patients, carers and clinicians. *Health Expectations* 24, 42–52 (2021).
107. Morgan, H., Thomson, G., Crossland, N., Dykes, F. & Hoddinott, P. Combining PPI with qualitative research to engage ‘harder-to-reach’ populations: Service user groups as co-applicants on a platform study for a trial. *Research Involvement and Engagement* 2, (2016).
108. Moodley, K. & Beyer, C. Tygerberg Research Ubuntu-Inspired Community Engagement Model: Integrating Community Engagement into Genomic Biobanking. *Biopreservation and Biobanking* 17, 613–624 (2019).
109. Mockford, C., Staniszewska, S., Griffiths, F. & Herron-Marx, S. The impact of patient and public involvement on UK NHS health care: A systematic review. *International Journal for Quality in Health Care* 24, 28–38 (2012).
110. Mitchell, S. J. *et al.* Ethics and patient and public involvement with children and young people. *ARCHIVES OF DISEASE IN CHILDHOOD-EDUCATION AND PRACTICE EDITION* 104, 195-+.
111. Mitchell, C. *et al.* Value and learning from carer involvement in a cluster randomised controlled trial and process evaluation - Organising Support for Carers of Stroke Survivors (OSCARSS). *Research Involvement and Engagement* 6, (2020).
112. Michelson, K. N. *et al.* The Process and Impact of Stakeholder Engagement in Developing a Pediatric Intensive Care Unit Communication and Decision-Making Intervention. *Journal of Patient Experience* 3, 108–118 (2016).
113. Miah, J. *et al.* Patient and Public Involvement for Dementia Research in Low- and Middle-Income Countries: Developing Capacity and Capability in South Asia. *Frontiers in Neurology* 12, (2021).
114. Miah, J. *et al.* Impact of involving people with dementia and their care partners in research: A qualitative study. *BMJ Open* 10, (2020).
115. Miah, J. *et al.* Patient and public involvement in dementia research in the European Union: A scoping review. *BMC Geriatrics* 19, (2019).
116. McNichol, E. Involving patients with leg ulcers in developing innovations in treatment and management strategies. *British Journal of Community Nursing* 19, S27–S32 (2014).
117. McMillan, B. *et al.* Using patient and public involvement to improve the research design and funding application for a project aimed at fostering a more collaborative approach to the nhs health check: The caviar project (better care via improved access to records). *Research Involvement and Engagement* 4, (2018).
118. McGoohan, K. *et al.* A Preliminary Investigation of the Views of People With Parkinson’s (With and Without Psychosis) and Caregivers on Participating in Clinical Trials During the Covid-19 Pandemic: An Online Survey. *Frontiers in Psychiatry* 11, (2020).
119. Mayland, C. R. *et al.* Assessing quality of care for the dying from the bereaved relatives’ perspective: Using pre-testing survey methods across seven countries to develop an international outcome measure. *Palliative Medicine* 33, 357–368 (2019).
120. Matthews, R. & Papoulias, C. Toward Co-productive Learning? The Exchange Network as Experimental Space. *Frontiers in Sociology* 4, (2019).
121. Matthews, R., Kaur, M., French, C., Baker, A. & Reed, J. How helpful are Patient and Public Involvement strategic documents - Results of a framework analysis using 4Pi National Involvement Standards. *Research Involvement and Engagement* 5, (2019).
122. Mathie, E. *et al.* Regional working in the east of England: Using the UK national standards for public involvement. *Research Involvement and Engagement* 4, (2018).
123. Mathie, E. *et al.* Reciprocal relationships and the importance of feedback in patient and public involvement: A mixed methods study. *Health Expectations* 21, 899–908 (2018).
124. Mathie, E. *et al.* The role of patient and public involvement leads in facilitating feedback: ‘invisible work’. *Research Involvement and Engagement* 6, (2020).
125. Masoud, S. S. *et al.* Engagement with a diverse Stakeholder Advisory Council for research in dementia care. *Research Involvement and Engagement* 7, (2021).
126. Martin, S. *et al.* Exploring attitudes and preferences for dementia screening in Britain: Contributions from carers and the general public. *BMC Geriatrics* 15, (2015).
127. Marks, S., Mathie, E., Smiddy, J., Jones, J. & Da Silva-Gane, M. Reflections and experiences of a coresearcher involved in a renal research study. *Research Involvement and Engagement* 4, (2018).
128. Mann, C., Chilcott, S., Plumb, K., Brooks, E. & Man, M. S. Reporting and appraising the context, process and impact of ppi on contributors, researchers and the trial during a randomised controlled trial - the 3d study. *Research Involvement and Engagement* 4, (2018).
129. Madden, M. *et al.* Producing co-production: Reflections on the development of a complex intervention. *Health Expectations* 23, 659–669 (2020).
130. Madden, M. & Morley, R. Exploring the challenge of health research priority setting in partnership: Reflections on the methodology used by the james lind alliance pressure ulcer priority setting partnership. *Research Involvement and Engagement* 2, (2016).
131. Maccarthy, J., Guerin, S., Wilson, A. G. & Dorris, E. R. Facilitating public and patient involvement in basic and preclinical health research. *PLoS ONE* 14, (2019).
132. Litherland, R. *et al.* Reflections on PPI from the ‘Action on Living Well: Asking You’ advisory network of people with dementia and carers as part of the IDEAL study. *Dementia* 17, 1035–1044 (2018).
133. Lidington, E. *et al.* Beyond Teenage and Young Adult Cancer Care: Care Experiences of Patients Aged 25–39 Years Old in the UK National Health Service. *Clinical Oncology* 33, 494–506 (2021).
134. Lessard, D., Engler, K., Toupin, I., Routy, J. P. & Lebouché, B. Evaluation of a project to engage patients in the development of a patient-reported measure for HIV care (the I-Score Study). *Health Expectations* 22, 209–225 (2019).
135. Leslie, M., Khayatzadeh-Mahani, A. & Mackean, G. Recruitment of caregivers into health services research: Lessons from a user-centred design study. *Research Involvement and Engagement* 5, (2019).
136. Lenze, E. J. *et al.* Older Adults’ Perspectives on Clinical Research: A Focus Group and Survey Study. *American Journal of Geriatric Psychiatry* 24, 893–902 (2016).
137. Lenguerrand, E. *et al.* Effect of Group-Based Outpatient Physical Therapy on Function After Total Knee Replacement: Results From a Multicenter Randomized Controlled Trial. *Arthritis Care and Research* 72, 768–777 (2020).
138. Leese, J. *et al.* Experiences of self-care during the COVID-19 pandemic among individuals with rheumatoid arthritis: A qualitative study. *Health Expectations* (2021).
139. Lee, C. *et al.* Getting underneath the skin: A community engagement event for optimal vitamin D status in an ‘easily overlooked’ group. *Health Expectations* 22, 1322–1330 (2019).
140. Law, J. *et al.* Decision-making for older patients undergoing emergency laparotomy: defining patient and clinician values and priorities. *Colorectal Disease* 22, 1694–1703 (2020).
141. Koniotou, M. *et al.* Involving older people in a multi-centre randomised trial of a complex intervention in pre-hospital emergency care: Implementation of a collaborative model. *Trials* 16, (2015).
142. Knowles, S. *et al.* Hidden caring, hidden carers? Exploring the experience of carers for people with long-term conditions. *Health and Social Care in the Community* 24, 203–213 (2016).
143. Kleme, J. *et al.* Patient perspective in health technology assessment of pharmaceuticals in Finland. *International Journal of Technology Assessment in Health Care* 30, 306–311 (2014).
144. Klaprat, N. M. D. *et al.* Filling gaps in type 1 diabetes and exercise research: A scoping review and priority-setting project. *BMJ Open Diabetes Research and Care* 8, (2020).
145. Kirwan, J. R. *et al.* Emerging Guidelines for Patient Engagement in Research. *Value in Health* 20, 481–486 (2017).
146. Kelly, S. *et al.* Dementia priority setting partnership with the James Lind Alliance: Using patient and public involvement and the evidence base to inform the research agenda. *Age and Ageing* 44, 985–993 (2015).
147. Kelly, L. E. *et al.* Innovative approaches to investigator-initiated, multicentre paediatric clinical trials in Canada. *BMJ Open* 9, (2019).
148. Kelemen, M., Surman, E. & Dikomitis, L. Cultural animation in health research: An innovative methodology for patient and public involvement and engagement. *Health Expectations* 21, 805–813 (2018).
149. Karlsson, P. *et al.* Stakeholder consensus for decision making in eye-gaze control technology for children, adolescents and adults with cerebral palsy service provision: findings from a Delphi study. *BMC Neurology* 21, (2021).
150. Kandiyali, R. *et al.* Working with Patients and Members of the Public: Informing Health Economics in Child Health Research. *PharmacoEconomics - Open* 3, 133–141 (2019).
151. Kaisler, R. E., Missbach, B. & Kaisler, R. E. Co-creating a patient and public involvement and engagement ‘how to’ guide for researchers. *Research Involvement and Engagement* 6, (2020).
152. Jull, J. *et al.* Engaging knowledge users in development of the CONSORT-equity 2017 reporting guideline: A qualitative study using indepth interviews. *Research Involvement and Engagement* 4, (2018).
153. Jull, J. *et al.* Taking an integrated knowledge translation approach in research to develop the CONSORT-Equity 2017 reporting guideline: An observational study. *BMJ Open* 9, (2019).
154. Jose, C. *et al.* We are the stakeholders with the most at stake": Scientific and autism community co-researchers reflect on their collaborative experience in the CONNECT project. *Research Involvement and Engagement* 6, (2020).
155. Jørgensen, C. R., Eskildsen, N. B. & Johnsen, A. T. User involvement in a danish project on the empowerment of cancer patients – experiences and early recommendations for further practice. *Research Involvement and Engagement* 4, (2018).
156. Jones, J. K. *et al.* Rapid Analgesia for Prehospital hip Disruption (RAPID): Findings from a randomised feasibility study. *Pilot and Feasibility Studies* 5, (2019).
157. Jones, J. *et al.* Reporting on patient and public involvement (PPI) in research publications: using the GRIPP2 checklists with lay co-researchers. *Research Involvement and Engagement* 7, (2021).
158. Jones, J. *et al.* Randomised feasibility study of prehospital recognition and antibiotics for emergency patients with sepsis (PhRASe). *Scientific Reports* 11, (2021).
159. Jones, H. *et al.* Co-creation and User Perspectives for Upper Limb Prosthetics. *Frontiers in Neurorobotics* 15, (2021).
160. Jones, E. L. *et al.* Quality of reporting on patient and public involvement within surgical research a systematic review. *Annals of Surgery* 261, 243–250 (2015).
161. Jokanovic, N. *et al.* Development of consumer information leaflets for deprescribing in older hospital inpatients: A mixed-methods study. *BMJ Open* 9, (2019).
162. Johnson, H. *et al.* Patient and public involvement in palliative care research: What works, and why? A qualitative evaluation. *Palliative Medicine* 35, 151–160 (2021).
163. Johnsen, A. T. *et al.* Conceptualizing patient empowerment in cancer follow-up by combining theory and qualitative data. *Acta Oncologica* 56, 232–238 (2017).
164. Jeyaraman, M. M. *et al.* Interventions and strategies involving primary healthcare professionals to manage emergency department overcrowding: A scoping review. *BMJ Open* 11, (2021).
165. Jewell, A. *et al.* The maudsley biomedical research centre (Brc) data linkage service user and carer advisory group: Creating and sustaining a successful patient and public involvement group to guide research in a complex area. *Research Involvement and Engagement* 5, (2019).
166. Jennings, H., Slade, M., Bates, P., Munday, E. & Toney, R. Best practice framework for Patient and Public Involvement (PPI) in collaborative data analysis of qualitative mental health research: Methodology development and refinement. *BMC Psychiatry* 18, (2018).
167. Jamal, Z. *et al.* Patient and public involvement prior to trial initiation: lessons learnt for rapid partnership in the COVID-19 era. *Research Involvement and Engagement* 7, (2021).
168. Isham, L., Bradbury-Jones, C. & Hewison, A. Reflections on engaging with an advisory network in the context of a ‘sensitive’ research study. *International Journal of Social Research Methodology* 22, 67–79 (2019).
169. Ingegnoli, F. *et al.* Relevant non-pharmacologic topics for clinical research in rheumatic musculoskeletal diseases: The patient perspective. *International Journal of Rheumatic Diseases* 23, 1305–1310 (2020).
170. Ingegnoli, F. *et al.* COVID-19 related poor mental health and sleep disorders in rheumatic patients: a citizen science project. *BMC Psychiatry* 21, (2021).
171. Ibsen, C. *et al.* “Keep it simple”: Perspectives of patients with low back pain on how to qualify a patient-centred consultation using patient-reported outcomes. *Musculoskeletal Care* 17, 313–326 (2019).
172. Hyde, C., Dunn, K. M., Higginbottom, A. & Chew-Graham, C. A. Process and impact of patient involvement in a systematic review of shared decision making in primary care consultations. *Health Expectations* 20, 298–308 (2017).
173. Hunt, A. *et al.* "Why does it happen like this?’ Consulting with users and providers prior to an evaluation of services for children with life limiting conditions and their families. *JOURNAL OF CHILD HEALTH CARE* 19, 320–333.
174. Hovén, E. & Eriksson, L. What makes it work? Exploring experiences of patient research partners and researchers involved in a long-term co-creative research collaboration. *Research Involvement and Engagement* 6, (2020).
175. Hoddinott, P. *et al.* How to incorporate patient and public perspectives into the design and conduct of research [version 1; peer review: 3 approved, 2 approved with reservations]. *F1000Research* 7, (2018).
176. Hernar, I. *et al.* Use of patient-reported outcome measures (PROMs) in clinical diabetes consultations: The DiaPROM randomised controlled pilot trial. *BMJ Open* 11, (2021).
177. Haywood, K., Lyddiatt, A., Brace-McDonnell, S. J., Staniszewska, S. & Salek, S. Establishing the values for patient engagement (PE) in health-related quality of life (HRQoL) research: an international, multiple-stakeholder perspective. *Quality of Life Research* 26, 1393–1404 (2017).
178. Haywood, K. *et al.* Patient and public engagement in health-related quality of life and patient-reported outcomes research: what is important and why should we care? Findings from the first ISOQOL patient engagement symposium. *Quality of Life Research* 24, 1069–1076 (2015).
179. Hayes, G., Costello, H., Nurock, S., Cornwall, A. & Francis, P. Ticking boxes or meaningful partnership – The experience of lay representation, participant and study partner involvement in Brains for Dementia Research. *Dementia* 17, 1023–1034 (2018).
180. Hawarden, A. *et al.* Public priorities for osteoporosis and fracture research: results from a focus group study. *Archives of Osteoporosis* 15, (2020).
181. Hausmann, J. S. *et al.* Immediate effect of the COVID-19 pandemic on patient health, health-care use, and behaviours: results from an international survey of people with rheumatic diseases. *The Lancet Rheumatology* 3, e707–e714 (2021).
182. Haugstvedt, A. *et al.* Nurses’ and physicians’ experiences with diabetes consultations and the use of dialogue tools in the DiaPROM pilot trial: A qualitative study. *Diabetic Medicine* 38, (2021).
183. Harris, L. K., Skou, S. T., Juhl, C. B., Jäger, M. & Bricca, A. Recruitment and retention rates in randomised controlled trials of exercise therapy in people with multimorbidity: a systematic review and meta-analysis. *Trials* 22, (2021).
184. Hancock, A., Weeks, A. D., Furber, C., Campbell, M. & Lavender, T. The Recognition of Excessive blood loss At ChildbirTh (REACT) Study: a two-phase exploratory, sequential mixed methods inquiry using focus groups, interviews and a pilot, randomised crossover study. *BJOG: An International Journal of Obstetrics and Gynaecology* 128, 1843–1854 (2021).
185. Hamilton, C. B., Leese, J. C., Hoens, A. M. & Li, L. C. Framework for Advancing the Reporting of Patient Engagement in Rheumatology Research Projects. *Current Rheumatology Reports* 19, (2017).
186. Hamilton, C. B. *et al.* Development and pre-testing of the Patient Engagement in Research Scale (PEIRS) to assess the quality of engagement from a patient perspective. *PLoS ONE* 13, (2018).
187. Hamilton, C. B. *et al.* An empirically based conceptual framework for fostering meaningful patient engagement in research. *Health Expectations* 21, 396–406 (2018).
188. Hall, C. L. *et al.* Consensus workshops on the development of an ADHD medication management protocol using QbTest: Developing a clinical trial protocol with multidisciplinary stakeholders. *BMC Medical Research Methodology* 19, (2019).
189. Hall, A., Boulton, E. & Stanmore, E. Older adults’ perceptions of wearable technology hip protectors: implications for further research and development strategies. *Disability and Rehabilitation: Assistive Technology* 14, 663–668 (2019).
190. Haenssgen, M. J., Charoenboon, N., Thavethanutthanawin, P. & Wibunjak, K. Tales of treatment and new perspectives for global health research on antimicrobial resistance. *Medical Humanities* (2020).
191. Haenssgen, M. J. New impulses from international development for more comprehensive and balanced public engagement evaluation. *Global Health Action* 12, (2019).
192. Gumm, R. *et al.* Improving communication between staff and disabled children in hospital wards: Testing the feasibility of a training intervention developed through intervention mapping. *BMJ Paediatrics Open* 1, (2017).
193. Grundy, A. *et al.* Public involvement in health outcomes research: Lessons learnt from the development of the recovering quality of life (ReQoL) measures. *Health and Quality of Life Outcomes* 17, (2019).
194. Gregory, S. *et al.* Research participants as collaborators: Background, experience and policies from the PREVENT Dementia and EPAD programmes. *Dementia* 17, 1045–1054 (2018).
195. Greenwood, K. *et al.* The impact of Patient and Public Involvement in the SlowMo study: Reflections on peer innovation. *Health Expectations* (2021).
196. Greenhalgh, T. *et al.* Frameworks for supporting patient and public involvement in research: Systematic review and co-design pilot. *Health Expectations* 22, 785–801 (2019).
197. Gray-Burrows, K. A. *et al.* Role of patient and public involvement in implementation research: A consensus study. *BMJ Quality and Safety* 27, 858–864 (2018).
198. Goodridge, D. *et al.* Building patient capacity to participate in care during hospitalisation: A scoping review. *BMJ Open* 9, (2019).
199. Gooberman-Hill, R. *et al.* Designing a placebo device: Involving service users in clinical trial design. *Health Expectations* 16, e100–e110 (2013).
200. Gonzalez, M. *et al.* Informing the development of the Canadian Neurodiversity Platform: What is important to parents of children with neurodevelopmental disabilities? *Child: Care, Health and Development* (2021).
201. Gonzalez, M. *et al.* Strategies used to engage hard-to-reach populations in childhood disability research: a scoping review. *Disability and Rehabilitation* 43, 2815–2827 (2021).
202. Frost, J., Gibson, A., Harris-Golesworthy, F., Harris, J. & Britten, N. Patient involvement in qualitative data analysis in a trial of a patient-centred intervention: Reconciling lay knowledge and scientific method. *Health Expectations* 21, 1111–1121 (2018).
203. Fox, G. *et al.* Patient engagement in preclinical laboratory research: A scoping review. *EBioMedicine* 70, (2021).
204. Foster, M. *et al.* Partnering with patients to get better outcomes with chimeric antigen receptor T-cell therapy: towards engagement of patients in early phase trials. *Research Involvement and Engagement* 6, (2020).
205. Forsythe, L. P. *et al.* Patient engagement in research: Early findings from the patient-centered outcomes research institute. *Health Affairs* 38, 359–367 (2019).
206. Forsyth, F., Saunders, C., Elmer, A. & Badger, S. ‘A group of totally awesome people who do stuff’ - a qualitative descriptive study of a children and young people’s patient and public involvement endeavour. *Research Involvement and Engagement* 5, (2019).
207. Fleming, P. R. *et al.* Patient engagement in fertility research: bench research, ethics, and social justice. *Research Involvement and Engagement* 7, (2021).
208. Finderup, J., Lomborg, K., Jensen, J. D. & Stacey, D. Choice of dialysis modality: patients’ experiences and quality of decision after shared decision-making. *BMC Nephrology* 21, (2020).
209. Finderup, J., Jensen, J. D. & Lomborg, K. Evaluation of a shared decision-making intervention for dialysis choice at four Danish hospitals: A qualitative study of patient perspective. *BMJ Open* 9, (2019).
210. Finderup, J., Crowley, A., Søndergaard, H. & Lomborg, K. Involvement of patients with chronic kidney disease in research: A case study. *Journal of Renal Care* 47, 73–86 (2021).
211. Fergusson, D. *et al.* The prevalence of patient engagement in published trials: A systematic review. *Research Involvement and Engagement* 4, (2018).
212. Farr, W. J. *et al.* Feasibility of a randomised controlled trial to evaluate home-based virtual reality therapy in children with cerebral palsy. *Disability and Rehabilitation* 43, 85–97 (2021).
213. Evans, D. *et al.* Extent, quality and impact of patient and public involvement in antimicrobial drug development research: A systematic review. *HEALTH EXPECTATIONS* 21, 75–81.
214. Evans, B. A., Porter, A., Snooks, H. & Burholt, V. A co-produced method to involve service users in research: The SUCCESS model. *BMC Medical Research Methodology* 19, (2019).
215. Evans, B. A. *et al.* Involving service users in trials: Developing a standard operating procedure. *Trials* 14, (2013).
216. Etkind, S. N., Lovell, N., Nicholson, C. J., Higginson, I. J. & Murtagh, F. E. M. Finding a ‘new normal’ following acute illness: A qualitative study of influences on frail older people’s care preferences. *Palliative Medicine* 33, 301–311 (2019).
217. Esmail, L., Moore, E. & Rein, A. Evaluating patient and stakeholder engagement in research: Moving from theory to practice. *Journal of Comparative Effectiveness Research* 4, 133–145 (2015).
218. Erridge, S. *et al.* A Comprehensive Patient and Public Involvement Program Evaluating Perception of Cannabis-Derived Medicinal Products in the Treatment of Acute Postoperative Pain, Nausea, and Vomiting Using a Qualitative Thematic Framework. *Cannabis and Cannabinoid Research* 5, 73–80 (2020).
219. Eltringham, S. A. *et al.* Experiences of dysphagia after stroke: An interview study of stroke survivors and their informal caregivers. *Geriatrics (Switzerland)* 4, (2019).
220. Edwards, M. *et al.* A classification of primary care streaming pathways in UK emergency departments: Findings from a multi-methods study comprising cross-sectional survey; site visits with observations, semi-structured and informal interviews. *International Emergency Nursing* 56, (2021).
221. e Wit, M. *et al.* Successful Stepwise Development of Patient Research Partnership: 14 Years’ Experience of Actions and Consequences in Outcome Measures in Rheumatology (OMERACT). *Patient* 10, 141–152 (2017).
222. Durand, H. *et al.* A qualitative comparison of high and low adherers with apparent treatment-resistant hypertension. *Psychology, Health and Medicine* 25, 64–77 (2020).
223. Duncan, E. *et al.* Guidance for reporting intervention development studies in health research (GUIDED): An evidence-based consensus study. *BMJ Open* 10, (2020).
224. Dreier, M., Baumgardt, J., Bock, T., Härter, M. & Liebherz, S. Development of an online suicide prevention program involving people with lived experience: ideas and challenges. *Research Involvement and Engagement* 7, (2021).
225. Dovey-Pearce, G., Walker, S., Fairgrieve, S., Parker, M. & Rapley, T. The burden of proof: The process of involving young people in research. *Health Expectations* 22, 465–474 (2019).
226. Doukani, A. *et al.* Towards a conceptual framework of the working alliance in a blended low-intensity cognitive behavioural therapy intervention for depression in primary mental health care: a qualitative study. *BMJ OPEN* 10, (2020).
227. Dipankui, M. T. *et al.* EVALUATION OF PATIENT INVOLVEMENT IN A HEALTH TECHNOLOGY ASSESSMENT. *International Journal of Technology Assessment in Health Care* 31, 166–170 (2015).
228. Dillon, E. C., Tuzzio, L., Madrid, S., Olden, H. & Greenlee, R. T. Measuring the Impact of Patient-Engaged Research: How a Methods Workshop Identified Critical Outcomes of Research Engagement. *JOURNAL OF PATIENT-CENTERED RESEARCH AND REVIEWS* 4, 237–246.
229. Dewey, R. S., Ward, C., Junor, A. & Horobin, A. Talk to us! Communication is a key factor in improving the comfort of MRI research participants. *Health Expectations* 24, 1137–1144 (2021).
230. Dewa, L. H. *et al.* Reflections, impact and recommendations of a co-produced qualitative study with young people who have experience of mental health difficulties. *Health Expectations* 24, 134–146 (2021).
231. Devan, H., Perry, M. A., Yaghoubi, M. & Hale, L. “A coalition of the willing”: experiences of co-designing an online pain management programme (iSelf-help) for people with persistent pain. *Research Involvement and Engagement* 7, (2021).
232. Dennehy, R., Cronin, M. & Arensman, E. Involving young people in cyberbullying research: The implementation and evaluation of a rights-based approach. *Health Expectations* 22, 54–64 (2019).
233. Deane, K. *et al.* Co-creation of patient engagement quality guidance for medicines development: An international multistakeholder initiative. *BMJ Innovations* 5, 43–55 (2019).
234. De Wit, M. *et al.* Building bridges between researchers and patient research partners: A report from the grappa 2014 annual meeting. *Journal of Rheumatology* 42, 1021–1026 (2015).
235. Dawson, S. *et al.* Patient and public involvement in doctoral research: Reflections and experiences of the PPI contributors and researcher. *Research Involvement and Engagement* 6, (2020).
236. Dawson, S., Campbell, S. M., Giles, S. J., Morris, R. L. & Cheraghi-Sohi, S. Black and minority ethnic group involvement in health and social care research: A systematic review. *Health Expectations* 21, 3–22 (2018).
237. Dawes, P., Newall, J., Stockdale, D. & Baguley, D. M. Natural history of tinnitus in adults: A cross-sectional and longitudinal analysis. *BMJ Open* 10, (2020).
238. Davies, M. A. M. *et al.* The consultation of rugby players in co-developing a player health study: Feasibility and consequences of sports participants as research partners. *Research Involvement and Engagement* 3, (2017).
239. Datta, S. *et al.* Assessing the cost-effectiveness of HPV vaccination strategies for adolescent girls and boys in the UK. *BMC Infectious Diseases* 19, (2019).
240. Cruz Rivera, S. *et al.* ‘Give Us the Tools!’: development of knowledge transfer tools to support the involvement of patient partners in the development of clinical trial protocols with patient-reported outcomes (PROs), in accordance with SPIRIT-PRO Extension. *BMJ Open* 11, (2021).
241. Crutch, S., Herron, D., Pickett, J., Rosser, S. & Rossor, M. Inspired by chance: valuing patients’ informal contributions to research. *BMJ-BRITISH MEDICAL JOURNAL* 371,.
242. Cruickshank, S. *et al.* Specialist breast cancer nurses’ views on implementing a fear of cancer recurrence intervention in practice: a mixed methods study. *Supportive Care in Cancer* 28, 201–210 (2020).
243. Cruice, M. *et al.* Creating a novel approach to discourse treatment through coproduction with people with aphasia and speech and language therapists. *Aphasiology* (2021).
244. Crudgington, H. *et al.* Core Health Outcomes in Childhood Epilepsy (CHOICE): Development of a core outcome set using systematic review methods and a Delphi survey consensus. *Epilepsia* 60, 857–871 (2019).
245. Crudgington, H. *et al.* Mapping epilepsy-specific patient-reported outcome measures for children to a proposed core outcome set for childhood epilepsy. *Epilepsy and Behavior* 112, (2020).
246. Crowe, S. *et al.* Are we asking the right questions? Working with the LGBTQ+ community to prioritise healthcare research themes. *Research Involvement and Engagement* 7, (2021).
247. Crocker, J. C. *et al.* Patient and public involvement (PPI) in UK surgical trials: A survey and focus groups with stakeholders to identify practices, views, and experiences. *Trials* 20, (2019).
248. Crocker, J. C., Boylan, A. M., Bostock, J. & Locock, L. Is it worth it? Patient and public views on the impact of their involvement in health research and its assessment: a UK-based qualitative interview study. *Health Expectations* 20, 519–528 (2017).
249. Creswell, C. *et al.* Cognitive therapy compared with CBT for social anxiety disorder in adolescents: A feasibility study. *Health Technology Assessment* 25, 1–93 (2021).
250. Cox, R., Kendall, M., Molineux, M., Miller, E. & Tanner, B. Consumer engagement in occupational therapy health-related research: A scoping review of the Australian Occupational Therapy Journal and a call to action. *Australian Occupational Therapy Journal* 68, 180–192 (2021).
251. Coupe, N. & Mathieson, A. Patient and public involvement in doctoral research: Impact, resources and recommendations. *Health Expectations* 23, 125–136 (2020).
252. Coulman, K. D. *et al.* Understanding and optimising patient and public involvement in trial oversight: An ethnographic study of eight clinical trials. *Trials* 21, (2020).
253. Corry, D. A. S. *et al.* Acceptability of a nurse-led, person-centred, anticipatory care planning intervention for older people at risk of functional decline: A qualitative study. *PLoS ONE* 16, (2021).
254. Coon, J. T. *et al.* End-user involvement in a systematic review of quantitative and qualitative research of non-pharmacological interventions for attention deficit hyperactivity disorder delivered in school settings: reflections on the impacts and challenges. *HEALTH EXPECTATIONS* 19, 1084–1097.
255. Combes, S., Gillett, K., Norton, C. & Nicholson, C. J. The importance of living well now and relationships: A qualitative study of the barriers and enablers to engaging frail elders with advance care planning. *Palliative Medicine* 35, 1137–1147 (2021).
256. Cho, Y. *et al.* Identifying patient-important outcomes in polycystic kidney disease: An international nominal group technique study. *Nephrology* 24, 1214–1224 (2019).
257. Cheung, P. P. *et al.* Recommendations for the involvement of Patient Research Partners (PRP) in OMERACT working groups. A report from the OMERACT 2014 working group on PRP. *Journal of Rheumatology* 43, 187–193 (2016).
258. Cavens, C., Imms, C., Drake, G., Garrity, N. & Wallen, M. Perspectives of children and adolescents with cerebral palsy about involvement as research partners: a qualitative study. *DISABILITY AND REHABILITATION*.
259. Casanova, T. *et al.* The impact of active research involvement of young children in the design of a new stereotest. *Research Involvement and Engagement* 6, (2020).
260. Carr, M. J. *et al.* Effects of the COVID-19 pandemic on primary care-recorded mental illness and self-harm episodes in the UK: a population-based cohort study. *The Lancet Public Health* 6, e124–e135 (2021).
261. Carr, E. C. J., Wallace, J. E., Pater, R. & Gross, D. P. Evaluating the relationship between well-being and living with a dog for people with chronic low back pain: A feasibility study. *International Journal of Environmental Research and Public Health* 16, (2019).
262. Carr, E. C. J. *et al.* Co-design of a patient experience survey for arthritis central intake: An example of meaningful patient engagement in healthcare design. *BMC Health Services Research* 19, (2019).
263. Cantwell, M. *et al.* The Development of the MedEx IMPACT Intervention: A Patient-Centered, Evidenced-Based and Theoretically-Informed Physical Activity Behavior Change Intervention for Individuals Living With and Beyond Cancer. *Cancer Control* 27, (2020).
264. Campbell, L. A., Lovas, D., Withers, E. & Peacock, K. Opening the door: Inviting youth and parent perspectives on youth mental health emergency department use. *Research Involvement and Engagement* 6, (2020).
265. Calvert, M. *et al.* SPIRIT-PRO Extension explanation and elaboration: guidelines for inclusion of patient-reported outcomes in protocols of clinical trials. *BMJ Open* 11, (2021).
266. Buck, D. *et al.* From plans to actions in patient and public involvement: qualitative study of documented plans and the accounts of researchers and patients sampled from a cohort of clinical trials. *BMJ OPEN* 4, (2014).
267. Brummell, Z., Vindrola-Padros, C., Braun, D. & Moonesinghe, S. R. NHS ‘Learning from Deaths’ reports: A qualitative and quantitative document analysis of the first year of a countrywide patient safety programme. *BMJ Open* 11, (2021).
268. Briggs, A. M. *et al.* The need for adaptable global guidance in health systems strengthening for musculoskeletal health: a qualitative study of international key informants. *Global Health Research and Policy* 6, (2021).
269. Briggs, A. M. *et al.* Health systems strengthening to arrest the global disability burden: Empirical development of prioritised components for a global strategy for improving musculoskeletal health. *BMJ Global Health* 6, (2021).
270. Brett, J. *et al.* Reaching consensus on reporting patient and public involvement (PPI) in research: Methods and lessons learned from the development of reporting guidelines. *BMJ Open* 7, (2017).
271. Brett, J. *et al.* Mapping the impact of patient and public involvement on health and social care research: A systematic review. *Health Expectations* 17, 637–650 (2014).
272. Brett, J. *et al.* A Systematic Review of the Impact of Patient and Public Involvement on Service Users, Researchers and Communities. *Patient* 7, 387–395 (2014).
273. Brereton, L. *et al.* Lay and professional stakeholder involvement in scoping palliative care issues: Methods used in seven European countries. *Palliative Medicine* 31, 181–192 (2017).
274. Breault, L. J. *et al.* People with lived experience (PWLE) of depression: Describing and reflecting on an explicit patient engagement process within depression research priority setting in Alberta, Canada. *Research Involvement and Engagement* 4, (2018).
275. Bray, N. *et al.* Powered mobility interventions for very young children with mobility limitations to aid participation and positive development: The empower evidence synthesis. *Health Technology Assessment* 24, 1–194 (2020).
276. Bray, L., Appleton, V. & Sharpe, A. ‘We should have been told what would happen’: Children’s and parents’ procedural knowledge levels and information-seeking behaviours when coming to hospital for a planned procedure. *Journal of Child Health Care* (2021).
277. Brand, S., Bramley, L., Dring, E. & Musgrove, A. Using patient and public involvement to identify priorities for research in long-term conditions management. *British Journal of Nursing* 29, 612–617 (2020).
278. Brainard, J. S. *et al.* Forced migrants involved in setting the agenda and designing research to reduce impacts of complex emergencies: Combining swarm with patient and public involvement. *Research Involvement and Engagement* 3, (2017).
279. Brady, L. M. *et al.* Involving young people in drug and alcohol research. *Drugs and Alcohol Today* 18, 28–38 (2018).
280. Boylan, A. M., Locock, L., Thomson, R. & Staniszewska, S. “About sixty per cent I want to do it”: Health researchers’ attitudes to, and experiences of, patient and public involvement (PPI)—A qualitative interview study. *Health Expectations* 22, 721–730 (2019).
281. Boutin, D. *et al.* Patient-partner engagement at the Centre de recherche du CHUS in the Province of Québec, Canada: from an intuitive methodology to outreach after three years of implementation. *Research Involvement and Engagement* 7, (2021).
282. Bourque, C. J. *et al.* The Integration of Resource Patients in Collaborative Research: A Mixed Method Assessment of the Nesting Dolls Design. *Patient Education and Counseling* 103, 1830–1838 (2020).
283. Boney, O. *et al.* Identifying research priorities in anaesthesia and perioperative care: Final report of the joint National Institute of Academic Anaesthesia/James Lind Alliance Research Priority Setting Partnership. *BMJ Open* 5, (2015).
284. Boivin, A. *et al.* Evaluating patient and public involvement in research. *BMJ (Online)* 363, (2018).
285. Boivin, A. *et al.* Patient and public engagement in research and health system decision making: A systematic review of evaluation tools. *Health Expectations* 21, 1075–1084 (2018).
286. Boden, C. *et al.* Patient partners’ perspectives of meaningful engagement in synthesis reviews: A patient-oriented rapid review. *Health Expectations* 24, 1056–1071 (2021).
287. Boateng, M. A. *et al.* Co-creation and prototyping of an intervention focusing on health literacy in management of malaria at community-level in Ghana. *Research Involvement and Engagement* 7, (2021).
288. Blackburn, S. *et al.* The extent, quality and impact of patient and public involvement in primary care research: A mixed methods study. *Research Involvement and Engagement* 4, (2018).
289. Blackburn, S. *et al.* Patient-reported quality indicators for osteoarthritis: A patient and public generated self-report measure for primary care. *Research Involvement and Engagement* 2, (2016).
290. Black, A. *et al.* What constitutes meaningful engagement for patients and families as partners on research teams? *Journal of Health Services Research and Policy* 23, 158–167 (2018).
291. Bisset, C. N. *et al.* Exploring shared surgical decision-making from the patient’s perspective: is the personality of the surgeon important? *Colorectal Disease* 22, 2214–2221 (2020).
292. Birkeland, S., Pedersen, S. S., Haakonsson, A. K., Barry, M. J. & Rottmann, N. Men’s view on participation in decisions about prostate-specific antigen (PSA) screening: Patient and public involvement in development of a survey. *BMC Medical Informatics and Decision Making* 20, (2020).
293. Bird, M. *et al.* Preparing for patient partnership: A scoping review of patient partner engagement and evaluation in research. *Health Expectations* 23, 523–539 (2020).
294. Bird, M. *et al.* A generative co-design framework for healthcare innovation: development and application of an end-user engagement framework. *Research Involvement and Engagement* 7, (2021).
295. Biggane, A. M., Olsen, M. & Williamson, P. R. PPI in research: A reflection from early stage researchers. *Research Involvement and Engagement* 5, (2019).
296. Biddle, M. S. Y., Gibson, A. & Evans, D. Attitudes and approaches to patient and public involvement across Europe: A systematic review. *Health and Social Care in the Community* 29, 18–27 (2021).
297. Bibi, R., Redwood, S. & Taheri, S. Raising the issue of overweight and obesity with the South Asian community. *BRITISH JOURNAL OF GENERAL PRACTICE* 64, 417–419.
298. Bethell, J. *et al.* Patient engagement in research related to dementia: A scoping review. *Dementia* 17, 944–975 (2018).
299. Berring, L. L., Buus, N. & Hybholt, L. Exploring the Dynamics of a Research Partnership in a Co-Operative Inquiry: A Qualitative Study. *Issues in Mental Health Nursing* 42, 818–826 (2021).
300. Bergmeier, H. J. *et al.* Global Health in Preconception, Pregnancy and Postpartum Alliance: Development of an international consumer and community involvement framework. *Research Involvement and Engagement* 6, (2020).
301. Beneciuk, J. M. *et al.* Musculoskeletal pain stakeholder engagement and partnership development: Determining patient-centered research priorities. *Research Involvement and Engagement* 6, (2020).
302. Bench, S., O’Shea, A. & Boaz, A. Patient and Family Member Experiences in Critical Care Research and Quality Improvement Projects. *Nursing Research* 69, 367–375 (2020).
303. Bench, S., Eassom, E. & Poursanidou, K. The nature and extent of service user involvement in critical care research and quality improvement: A scoping review of the literature. *International Journal of Consumer Studies* 42, 217–231 (2018).
304. Bedwell, C. & Lavender, T. Giving patients a voice: implementing patient and public involvement to strengthen research in sub-Saharan Africa. *JOURNAL OF EPIDEMIOLOGY AND COMMUNITY HEALTH* 74, 307–310.
305. Batchelor, J. M. *et al.* Home-based narrowband UVB, topical corticosteroid or combination for children and adults with vitiligo: HI-light vitiligo three-arm RCT. *Health Technology Assessment* 24, 1–164 (2020).
306. Banner, D. *et al.* Patient and public engagement in integrated knowledge translation research: Are we there yet? *Research Involvement and Engagement* 5, (2019).
307. Bajuaifer, S., Grey, M. J., Hancock, N. & Pomeroy, V. M. User perspectives on the design and setup of lower limb mirror therapy equipment after stroke: a technical report. *Physiotherapy (United Kingdom)* 113, 37–43 (2021).
308. Bailey, S., Boddy, K., Briscoe, S. & Morris, C. Involving disabled children and young people as partners in research: A systematic review. *Child: Care, Health and Development* 41, 505–514 (2015).
309. Avery, M. Consulting a young person’s advisory group: Planning healthcare research with and for young people with medically unexplained symptoms. *JOURNAL OF PSYCHOSOMATIC RESEARCH* 124,.
310. Arnstein, L. *et al.* Patient involvement in preparing health research peer-reviewed publications or results summaries: A systematic review and evidence-based recommendations. *Research Involvement and Engagement* 6, (2020).
311. Andrews, L. M., Allen, H., Sheppard, Z. A., Baylis, G. & Wainwright, T. W. More than just ticking a box…how patient and public involvement improved the research design and funding application for a project to evaluate a cycling intervention for hip osteoarthritis. *Research Involvement and Engagement* 1, (2015).
312. Alderson, H., Brown, R., Smart, D., Lingam, R. & Dovey-Pearce, G. ‘You’ve come to children that are in care and given us the opportunity to get our voices heard’: The journey of looked after children and researchers in developing a Patient and Public Involvement group. *Health Expectations* 22, 657–665 (2019).
313. Alahmadi, A. *et al.* An explainable algorithm for detecting drug-induced QT-prolongation at risk of torsades de pointes (TdP) regardless of heart rate and T-wave morphology. *Computers in Biology and Medicine* 131, (2021).
314. Al-Janabi, H. *et al.* Patient and Public Involvement (PPI) in Health Economics Methodology Research: Reflections and Recommendations. *Patient* 14, 421–427 (2021).
315. Abrehart, N. *et al.* “A little (PPI) MAGIC can take you a long way” : involving children and young people in research from inception of a novel medical device to multi-centre clinical trial Roald Dahl, James and the Giant Peach (1961). *Research Involvement and Engagement* 7, (2021).
316. Abayomi, J. C. *et al.* A patient and public involvement investigation into healthy eating and weight management advice during pregnancy. *International Journal for Quality in Health Care* 32, 28–34 (2020).

## Abbreviations

GRIPP: Guidance for Reporting the Involvement of Patients and the Public
PRISMA: Preferred Reporting Items for Systematic Reviews and Meta-analyses
NIHR: National Institutes for Health and Care Research
CIHR: Canadian Institutes of Health Research
OSSU: Ontario SPOR SUPPORT Unit
SPOR: Strategies for Patient-Oriented Research
